# Implementation of Point-of-Care PCR-testing for the diagnosis of respiratory infections in vulnerable patient populations

**DOI:** 10.1371/journal.pone.0307621

**Published:** 2025-07-29

**Authors:** Hannah Tolle, Jonas Wachinger, María del Mar Castro, Ivonne Morales, Claudia M. Denkinger

**Affiliations:** 1 Department of Infectious Diseases and Tropical Medicine, Center of Infectious Diseases, Heidelberg University Hospital, Heidelberg, Germany; 2 Heidelberg Institute of Global Health (HIGH), Heidelberg University Hospital, Heidelberg, Germany; 3 German Centre for Infection Research (DZIF), Partner Site Heidelberg University Hospital, Heidelberg, Germany; The University of Sydney, AUSTRALIA

## Abstract

**Background:**

Point-of-Care (POC) PCR-testing provides accurate, and timely results in diagnosing respiratory viral infections. Despite these benefits, stakeholder perceptions and its potential for improving health outcomes remain insufficiently explored. Therefore, we aim to explore the acceptability and feasibility of POC PCR-testing implementation in settings attended by vulnerable populations.

**Methods:**

We conducted semi-structured interviews with stakeholders from ambulatory settings, including decision makers, molecular diagnostics experts, healthcare workers, and patients. Study settings included two emergency departments (one adult, one pediatric), two oncology units, and two dialysis units, including both POC PCR implementors and non-implementors. We thematically analyzed our data, drawing on components of the Thematic Framework of Acceptability, the CFIR, and the Consolidated Framework for Sustainability Constructs in Healthcare.

**Results:**

Stakeholders recognized COVID-19, influenza A&B, and RSV as significant healthcare challenges and generally viewed POC PCR-testing as fit to address them. Stakeholders exhibited varying levels of knowledge about POC PCR-testing, with lower levels among non-implementors. While perceived benefits included rapid results, accuracy, and automation, concerns regarding cost, workload, and device throughput remained. Stakeholders recognized testing’s clinical impact in terms of transmission prevention and patient management. Perceived infection risk, disease prevalence, governmental regulations, and funding availability further influenced implementation decisions. Implementation processes were deemed straightforward, but limited involvement in decision-making processes dissatisfied some healthcare providers. End-users valued POC PCR-devices’ ease-of-use, while molecular diagnostics experts stressed that testing should be performed by medical staff. Sustainability considerations emphasized the stepwise development of testing guidelines, adaptation to local workflows, and continuous evaluation and quality control.

**Conclusion:**

POC PCR-testing for respiratory viral infections is generally accepted. Knowledge, cost, workload and perceived benefit of testing guide decisions, while testing upon suspicion is favored over screening strategies due to per-test-cost and low device throughput. Sustainability requires cost-efficiency through guidelines and outcome monitoring. While test accuracy and turnover-time are valued, clinical impact requires further investigation.

## Introduction

Respiratory viral infections contribute significantly to both morbidity and deaths, particularly in vulnerable patients [1]. Furthermore, unspecific early symptoms make the differentiation of potentially severe infections such as influenza, respiratory syncytial virus (RSV), and severe acute respiratory syndrome coronavirus 2 (SARS-CoV-2) from a common cold complicated [2]. Therefore, ensuring adequate patient management necessitates a timely, accurate, and reliable diagnosis [3–5]. The timeliness of diagnosis to inform individual therapy or infection control is especially important when dealing with patients marked by co-morbidities like immunosuppression or renal failure in congregate settings [6]. Besides an improvement in clinical decision-making and prevention of nosocomial transmission, an early diagnosis can protect clinical staff and thus avoid disruptions to the clinical workflow [7]. Ambulatory settings stand out as environments where large numbers of patients with diverse clinical profiles regularly intersect with one another and health care professionals [8,9].

Point of Care (POC) tests are conducted at or near the site of patient care and can achieve fast and accurate diagnoses [10]. They may complement traditional laboratory-based molecular testing methods, such as reverse transcription polymerase chain reaction (RT-PCR), due to the faster turn-around time of results and on-site detection, thus expediting the diagnostic process [11]. POC-tests are available for a range of respiratory viral infections, including RSV, influenza A&B, and COVID-19. They detect pathogens by amplifying viral nucleic acid (molecular-based) or by immunological detection of viral proteins (non-molecular based) [12]. Notably, in the case of COVID-19, antigen-detection rapid diagnostic tests (Ag-RDTs) have been widely employed in Europe [13] due to their affordability and rapid turnaround time. These advantages, however, come with lower diagnostic accuracy, a characteristic feature of non-molecular-based POC-tests [14,15].

To bridge this gap in accuracy, molecular-based POC-tests (POC PCR) have recently emerged for diagnosing respiratory viral infections [16]. For instance, compared to Ag-RDTs, molecular-based POC PCR-tests for SARS-CoV-2 boast higher test accuracy, while they offer a shorter time-to-diagnosis (~13 min) than to conventional RT-PCR-tests [14]. An assessment of influenza POC PCR-tests’ clinical performance found positive percent agreements with RT-PCR between 79 and 96% for influenza A and B, depending on the device used [17]. Similar results were observed for RSV POC PCR-testing in a pediatric ambulatory setting [18]. With the increasing availability of POC PCR and panel-tests, stakeholders’ and patients’ views regarding the tests’ feasibility and acceptability in the ambulatory sector need to be understood [19,20], as they are crucial to inform decisions on implementation and ensuring efficient and sustainable implementation processes [21]. However, such insights into stakeholder perceptions of POC PCR-tests in ambulatory settings remain limited. Thus, this study aims to generate in-depth, qualitative insights into the acceptability and feasibility of POC PCR-testing for diagnosing respiratory viral infections in settings attended by vulnerable patient groups.

## Methods

### Study settings

We conducted a qualitative study across multiple centers. Six facilities attended by high-risk patients were selected: two ambulatory oncology units, two ambulatory dialysis units, and two emergency departments (one adult and one pediatric) in southern Germany. Table 1 depicts study settings and their characteristics. Out of the eight study sites, three (two emergency care departments (E&P) and one dialysis unit (D1)) were affiliated to a large tertiary hospital and currently implement POC PCR-tests for respiratory viral infections. Standard testing steps of the used POC PCR-devices, as employed by the sites, are illustrated in S1. These three settings had an interdisciplinary task force, advised by molecular diagnostic experts, as the main decision-maker and a central cost center for financing decisions. Senior physicians and nurse managers were heads of department with a team of approximately 25–40 healthcare workers (HCWs).

**Table 1:** Characteristics of the interview settings and POC PCR-tests used before and during interviews.

Setting	Type of organization	Decision-making structure	Available Funding	Viruses tested	POC PCR-tests used
Emergency Department (E)	Tertiary hospital	Heads of departments, care managers, laboratory staff and task force	Cost center of university hospital (remuneration by health insurance providers and taxes)	COVID-19	Abbott ID Now (Abbott)
Pediatric Emergency Care Unit (P)	Tertiary hospital	Heads of departments, care managers, laboratory staff and task force	Cost center of university hospital (remuneration by health insurance providers and taxes)	COVID-19, RSV	Abbott ID Now (Abbott)
Dialysis Unit 1 (D1)	Tertiary hospital	Heads of departments, care managers, laboratory staff and task force	Cost center of university hospital (remuneration by health insurance providers and taxes), research projects	COVID-19, Paneltest^1^	Rhonda PCR-Schnelltest-system (Spindiag)
Dialysis Unit 2 (D2)	Ambulatory medical care center	Medical directors under a holding company, centralized management	Remuneration by health insurance providers, investments of holding company	None	None
Ambulatory Oncology Unit (Chemo) 1 (O1)	Ambulatory medical care center	Medical directors under a holding company, centralized management	Remuneration by health insurance providers, investments of holding company	None	None
Ambulatory Oncology Unit (Chemo) 2 (O2)	Ambulatory medical care center	Medical directors under a holding company, centralized management	Remuneration by health insurance providers	None	None

^1^Panel test of COVID-19, Influenza A&B and RSV.

^2^Experience with other tests was mentioned but not specified.

The two oncology units (O1&O2) and one dialysis unit (D2) were ambulatory medical care centers and were still considering implementation of POC PCR-testing. In these settings a set of medical directors under a holding company with centralized management supervised a team of 10–30 HCWs and physician assistants (Table 1). For advice on molecular diagnostics they relied on external laboratory groups.

### Recruitment and data collection

For the selection of interviewees, we followed the Consolidated Framework of Implementation Research (CFIR) defining stakeholders as persons who influence the outcome of implementation efforts [22]. We therefore recruited patients (or, in the pediatric emergency care unit, the legal guardian) (*P*; n = 20; mean interview duration: 17 min), HCWs (*H*; n = 9; mean interview duration: 25 min), and decision-makers (*D*; n = 9; mean interview duration: 33 min). Additionally, two molecular diagnostics experts, one who advises POC PCR-testing in a tertiary hospital (*L*1; interview duration 52 min), and one from a laboratory organization catering to ambulatory medical care centers (*L*2; interview duration 45 min), were interviewed. Recruitment took place via stratified purposeful snowball or opportunity sampling [23] in our study settings. Participants were approached via e-mail, telephone or in person in the respective settings. More recruitment details are illustrated in S7. In the three settings already using POC PCR-tests, we only included stakeholders who had first-hand experience with their use. The study enrolled a total of 40 individuals over the age of 18 between 21/10/2022 and 02/03/2023; sample sizes were informed by saturation considerations [24]. This study was approved by the ethics committee of the Heidelberg medical faculty (S-596/2022). Written informed consent was obtained for all participants. For more detailed information on the interviewed stakeholders see S2.

The lead author (HT), a medical professional, used a semi-structured guide (see S3) to conduct qualitative key-informant interviews [25] in German (n = 39 interviews, first language of both interviewer and respondent) and in English (n = 1 interview, second language of interviewer and first language of respondent). All interviews were conducted in a one-on-one, in person format in quiet areas at the point of care. Following informed consent, interviews were recorded and transcribed verbatim using an automated transcription tool [26] and corrected by HT, following a transcription protocol. Two interviewed patients objected to voice recording; detailed notes were taken during their interviews and memory transcripts were written immediately after. The interviewer took detailed fieldnotes after each interview, focusing on predominant themes, participant behavior and setting specifics.

### Data analysis

Informed by the Framework approach [27] to qualitative content analysis (see S4), HT familiarized herself with the data.

Based on this familiarization, HT revisited the literature and identified the Consolidated Framework for Implementation Research (CFIR) [28] as a starting point to explore factors influencing POC PCR-testing implementation. The CFIR condenses various models into five domains focusing on barriers and facilitators to implementation: [1] intervention characteristics, [2] inner and [3] outer settings, [4] individual characteristics, and [5] implementation process. Relevant constructs were identified through consensus decision-making and operationalized for our research purposes. For sustained used, we extended them with constructs from the Consolidated Framework for Sustainability Constructs in Healthcare [28] and summarized the resulting codebook under the term feasibility. An important base of successful implementation is the acceptability of the intervention as perceived by the stakeholders [21]. The CFIR focuses on the organizational perspective, while only partly considering individual perspectives. Therefore, we separately analyzed acceptability among stakeholders using the Theoretical Framework of Acceptability (TFA) [29]. It condenses factors related to acceptability of healthcare interventions into seven component constructs (affective attitude, burden, ethicality, intervention coherence, opportunity costs, perceived effectiveness, self-efficacy). These modifications partly align with updates in the 2022 version of CFIR [22]. We developed two distinct codebooks for themes related to acceptability and feasibility (S5) and indexed the data combining deductive and inductive approaches using NVivo 13 (2020). We repeated coding for all data after clarifications in code definitions were made before charting and mapping the data. The resulting coding trees for acceptability and feasibility are included in S6.

During these processes regular debriefings were held with researchers experienced in qualitative research (MC, JW) to mitigate researcher bias [30]. See S7 for a detailed statement of reflexivity.

## Results

We identified distinct steps required for implementing POC PCR-testing, as well as factors influencing success and sustained use. To highlight the influence of POC PCR-testing acceptability and feasibility, and to guide potential future implementation practices, we present our results structured into the necessary implementation and sustained use steps. They were largely shared across settings and stakeholders and include the choice and purchase of testing devices, delegation of tasks, logistic and technical setup, staff training, and regular quality assurance practices. We derived these steps from stakeholders’ descriptions of the ideal implementation process, their reported experiences, ideally involved stakeholders, and suggestions for improvement.

Table 2 describes the steps of implementation and sustained use, involved stakeholders, conditions perceived as necessary for implementation success, as well as reported issues and recommendations for the implementation of POC PCR-testing.

**Table 2:** Factors and Steps in Implementing and Sustaining POC PCR-testing reported in settings using POC PCR-testing.

Steps of implementation & sustained use	Stakeholders involved	Necessary conditions for implementation	Perceived feasibility	Reported issues and suggested changes
Implementation				
Information, decision to implement and choice of POC PCR-testing device	Task force, laboratory staff, heads of departments, principal investigators	Active access to manufacturer information and evidence (through laboratory staff/ contact with POC PCR producers)	Manageable due to opinion leaders (laboratory staff) and previous experience with producers	Lack of available options in the early phases of POC PCR-testing for respiratory diseases
Financing decisions and purchasing of devices	Cost center, research projects	Funding availability, research cooperations and remuneration conditions	Process as black box with little involvement of ambulatory units	Refusal of cost center to pay, availability of supplies
Logistic setup	Care management, laboratory technicians	Waiting room, space for testing device & dirty work area, regular material orderings and stockage, waste disposal	Needed structures already in place for other POC diagnostics	Increased away-time and time effort due to distant device location. Suggested closer affiliation of device to the unit
Technical integration	Producer, hospital technicians	Integration with hospital information system (HIS) for result documentation	Technically feasible (via interface), compatibility with standard HIS	High cost of interface
Delegation of tasks and responsibilities	Heads of departments	Clear accountability of roles	High willingness to participate and volunteered adoption of tasks	Opposition of some HCWs, wish for more inclusion of medical personnel in decision-making
Training and capacity building	Manufacturer customer service, medical devices officer	Initial training in handling of devices and documentation, scale up via snowball system	Perceived as practical, efficient and fast due to easy-to-use devices	Concern about passed-on handling mistakes, wish for follow-up trainings
Design of standard operating procedures (SOPs) or clinical guidelines	Heads of department, care management, hygiene experts	Stepwise creation of SOPs to consider clinical realities and infectious state, prevention of program drift	Easy design and adjustment of testing strategies according to prevalence and practicability	Fast changing conditions (prevalence, isolation, admission rules), need for flexibility of SOPs, wish for more involvement of medical personnel
Sustained use				
Quality control	Appointed laboratory staff, HCWs, official round-robin tests	Surveillance of correct test execution, confirmation of results via RT-PCR, device-internal calibration mechanisms, round-robin tests	Easily executable for trained staff, mostly automated processes in line with standard quality control measures	Long duration of calibration for new batch of cartridges, increased workload through confirmatory “double testing” (*H*, E), device malfunctions upon use by non-trained staff
Feedback and evaluation	Appointed laboratory staff, care managers, HCWs	Feedback through appointed laboratory staff, evaluation of clinical performance through task force	Mostly performed by external stakeholders	No fixed feedback structure, wish for more continuous evaluation of handling problems and discourse with involved staff
Intervention adaptation	Heads of department, care management, hygiene experts	Adaptation of viruses tested and testing indication according to need, identification of handling difficulties	Easily manageable through changing of SOPs and follow-up trainings	Not mentioned

In this section, we describe factors that influence stakeholders’ perceptions on feasibility and acceptability. Detailed results of our separate analysis for acceptability and feasibility can be found in S8 and S9. The used quotes are listed in their original language in S10.

## The Implementation of POC PCR-Testing for respiratory viral infections

### Information, decision to implement, and choice of POC PCR-testing device

#### Access to information and knowledge.

Respondents reported a good basis of evidence on POC PCR-test accuracy, while perceiving evidence on clinical impact as scarce. Decision-makers obtained knowledge from POC PCR producers, research projects, colleagues, medical journals, or laboratory staff. Decision-makers from tertiary hospitals were often informed actively about the possibility of POC PCR-testing through task forces or laboratory staff, while in the medical care centers information about POC PCR-testing foremost originated from reading or via hearsay from colleagues working in the tertiary care sector. The depth of scientific knowledge regarding the testing varied greatly among stakeholders. Knowledge was particularly low in environments where POC PCR-testing was not in use. Some stakeholders, including patients, expressed a high perceived need for more outcome-related evidence such as number-needed-to-treat, prevention of transmission, and evidence on secondary cost savings for the healthcare system as well as an assessment of necessary workflows in non-university settings. This overall rather low intervention coherence affected acceptability, especially in medical care centers, where stakeholders with less knowledge or more misconceptions about POC PCR-testing tended to be more skeptical.

#### POC PCR implementation decision.

The implementation decision and device selection involved centralized decision-making with limited end-user input. A healthcare worker noted, “*we knew [the test] was coming about maybe two, three weeks before. But by then it was already a done deal. So, there wasn’t really much that we could have done about it*” (*H*, E). Decisions on implementation sites were termed “*institutional political decisions*” in tertiary hospitals (*L*1).

Considerations influencing the overall implementation decision included the perceived relative advantage and effectiveness of POC PCR-testing, the tension for change in the pre-implementation situation, and the anticipated testing burden.

All stakeholders highlighted advantages like short time-to-result, high accuracy, low personnel-binding, and non-stop availability: “*Anyone can access it at any time*” (*H*, E). The perceived clinical impact and effectiveness varied, with patients focusing on risk of infection and transmission prevention, as “*here in the room we are quite close together*” (*P*, P), and healthcare providers valuing the ability for targeted patient management, as “*we simply can’t make 42 beds into 20 because everyone [with unclear infection status] is isolated*” (*D*, D1).

POC PCR-testing also was perceived as alleviating the burden of potential false-positive Ag-RDTs, for example the need to reschedule appointments: “*The patient does not have to go back home, make a new appointment, organize everything all over again*” (*H*, P). Similarly, for healthcare providers in medical care centers, POC-PCR availability greatly facilitated decisions on chemotherapy continuation for patients with unclear respiratory symptoms: *“You have the result and you can decide, chemo yes or no*” (*D*, O2). These factors positively affected perceived effectiveness and therefore acceptability of testing. Concerns affecting acceptability included the burden in form of cost and workload, as well as limitations to perceived effectiveness through lack of viral quantification, and low device throughput (1–5 tests per runtime). While patients acknowledged discomfort with nasal swabs, particularly in children, testing was generally considered as a minor inconvenience with low perceived burden. Furthermore, some patients highlighted the shortened isolation as a benefit, while others noted the negative consequences of a positive test result (isolation, stigma, fear of postponed other diagnostics) as a perceived burden. Patients’ affective attitude was influenced by a feeling of security through implemented testing and responsibility towards other patients, while one patient mentioned pandemic fatigue and reluctance due to feeling monitored and incapacitated.

Concerning the affective attitude, some stakeholders expressed fatigue about COVID-19 testing and that they “*don’t want to know*” (*D*, P) the results due to challenging consequences for patient management. Central laboratory staff was partly perceived as opposing POC PCR implementation in tertiary hospitals: “*Probably more for political reasons, yes, to ensure a certain minimum standard of care, but also to keep the focus on the laboratory*” (*L*1).

The tension for change and the perceived need for improved diagnostics were notably shaped by past experiences, particularly the ongoing COVID-19 pandemic at the time of data collection. Stakeholders’ evaluation of patient risk as well as patients’ assessments of their own vulnerability played a crucial role, leading to a fear of transmission: “*There are very sick patients here who have a super high risk. And for them, the virus can also be absolutely fatal*” (*P*, D1). Some medical personnel considered improved testing strategies as “*indispensable*” (*D*, O2) or “*life-saving*” (*D*, O1) in vulnerable populations, citing exposure and consecutive absences, infection, and challenges in handling patients amid delays in RT-PCR results. The alleviation of fears increased the perceived ethicality and positive affective attitude towards testing, affecting acceptability. Instances like a patient with unclear symptoms testing negative with an Ag-RDT but turning positive in PCR results highlighted challenges.

Stakeholders noted the inadequacy of POC-testing alternatives, such as external testing, ventilation, and spatial constraints, which had helped them to get through the pandemic “*rather poorly than well*” (*D*, O2). Due to extended exposure in chemo or dialysis rooms, stakeholders in medical care centers considered an alternative strategy of isolation only upon suspicion as unfeasible: “*The clinician will not isolate the patient until he has a result*” (*L1*), while patients reported the testing alternatives (continued wearing of FFP2 masks, room ventilation in winter) as a burden. Patient admission requirements in other health institutions, such as a negative COVID-19 PCR-test result, served as an additional incentive for implementation in tertiary hospitals.

In contrast, some stakeholders in settings without POC PCR-testing saw alternatives like basic hygiene and Ag-RDTs as sufficient for COVID-19 and influenza control: “*I suppose everything has worked out fine so far; why change much now?”* (*P*, D1). Testing for influenza was not seen as worthwhile, except for vulnerable groups. “*If I only have outpatients and no intensive care or hematological patients, it’s possible to manage without [POC PCR-testing]*” (*L*2). Some stakeholders expected limited impact and effectiveness of POC PCR implementation on managing outbreaks, stating, “*[It] probably wouldn’t be of much use*” (*D*, O1).

Financial considerations, lack of personnel, and time constraints were main arguments against POC PCR implementation in all settings. Medical personnel anticipated opposition, particularly from HCWs concerned about the burden of additional workload and disruptions in workflows, leading to concerns that “*the reception from the (laughs) colleagues is always rather, yes, not so good*“(*H*, P). One decision-maker found the effort unmanageable, stating, “*It is another additional personnel effort requiring people that I don’t have*” (*D*, O1). However, some stakeholders suggested mitigation of this burden through secondary time savings as a consequence of targeted patient management and available support options. Perceived burdens included unnecessary double testing with both POC and RT-PCR, lengthy validation processes, and the significant “*cumulative burden*” (*H*, P) with other COVID-19-related measures. One healthcare worker highlighted the challenge of managing tests during busy periods, noting, “*When at noon the emergency room is completely full... it’s not the test or the invention itself that’s the problem, but simply because you know or think, wow, now I have to do this as well*” (*H*, E). Stakeholders, however, did not perceive the test to be interfering with standard patient care, evaluating the opportunity cost as low. Anticipated time-saving measures involved using panel tests and widespread POC PCR implementation (eliminating testing for other departments) and relying on POC PCR results without subsequent RT-PCR confirmation. “*So basically, if this was taken as the standard and then not sent to the laboratory at all, it would definitely save time and money*” (*H*, P).

#### Choice of device.

The choice of testing device in implementing settings was made pragmatically in consultation with laboratory delegates, influenced by cost considerations, previous experience with different producers, and, in the early phases of the COVID-19 pandemic, limited availability of devices, reagents and cartridges.

### Financing decisions and purchasing of the device

For decision-makers, the financial burden in the form of cost of POC PCR-testing and remuneration considerations crucially affected acceptability. Concerns included the purchase of the POC PCR-device, the higher cost per test compared to laboratory testing, and potential overuse: “*You have to think economically. You have a responsibility towards the welfare state*” (*L*2). Tertiary hospitals based financing decisions on their institution’s cost center. External incentives included financing testing devices within research projects: “*The device is still a cost-neutral loan at the moment*” (*D*, D1).

Decision-makers, especially in medical care centers, worried about reimbursement difficulties with health insurance providers: “*That’s the other question, whether there’s reimbursement, what’s paid for by health insurance. Probably the purchase of devices. Less likely for material expenditure*” (*D*, D2). They also emphasized a lack of funding through institutional administrations: “*So there... yes, our politics or our health care system is not so consistent about what they want. Do they want health, or do they want money?”* (*D*, D1).

### Logistic setup and technical integration

Medical personnel identified infrastructure requirements for POC PCR-testing to encompass well-ventilated testing rooms, patient flow segregation and possible isolation, proximity of dedicated testing areas for separation from direct patient care, designated storage rooms, and a system for infectious waste disposal. Their compatibility with POC PCR-testing within the current facilities was perceived as a straightforward addition in all settings. In one dialysis unit, the ideal of separate testing and reality deferred, as one decision-maker noted: “*So ideally testing in a special room, [...] and in reality, in a majority of cases, we test during dialysis*” (*D*, D1). Technical integration of devices into the settings’ information system with automated digital result transfer was possible, albeit expensive, leading to some settings to opt for manual documentation.

### Delegation of tasks and responsibilities

**Fig 1:**
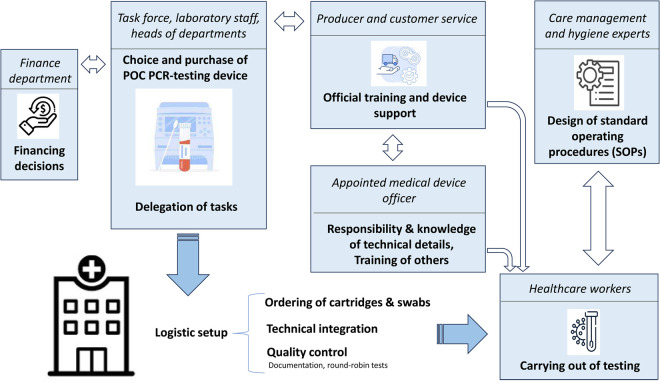
Responsibilities of stakeholders in the implementation process in ambulatory care units associated with a tertiary hospital.

Stakeholders saw delegated roles as crucial for facilitating implementation, with specific tasks assigned by heads of departments or medical directors. *“[Clear responsibilities] are important due to interdisciplinarity, also due to coordination, in order to streamline work processes and also to use personnel resources, yes, effectively*” (*D*, P). The exemplary responsibilities of different stakeholders in the tertiary hospitals in the implementation process are depicted in Fig 1.

Laboratory staff, acting as opinion leaders, played a key role, and most settings appointed a medical devices officer who received initial training from the manufacturer and a license to train others. This officer should be “*[...]well versed in the device and also knows the materials, how to use it, and of course also has contacts to customer service if something doesn’t work*” (*D*, P).

In the tertiary hospitals, HCWs were engaged through an official short announcement and their involvement varied from passive compliance to active contribution and wish for greater involvement in the intervention design. HCWs perceived the POC PCR-devices themselves as “*fool-proof*” and “*self-explanatory*” (*H*, P), expressing high levels of self-efficacy. Concerning ethicality and affective attitude, many HCWs showed a high willingness to take on additional workload and hygiene measures for the wellbeing of patients. “*Of course, this means working extra hours. That is how it is*” (*H*, E).

Molecular diagnostics experts underscored the significance of conducting tests with “*medically trained staff*” (*L*2) who possess adequate hygiene training, emphasizing that involving unqualified helpers was “*no-go*” (*L*2).

### Training and capacity building

In all settings stakeholders stated that a core group of HCWs should receive initial training from the manufacturer, who then trained colleagues in a snowball system. Stakeholders who had already implemented POC PCR-testing evaluated this approach as practical and sufficient to “*feel confident*” (*H*, P) about procedures and documentation. Some expressed a need for more official follow-up training to avoid passing on sources of error, and better access to scientific background knowledge through official sources. “*I could just imagine, that if there are any sources of error, that of course that is then constantly passed on. If one is trained [by snowball system]. I think it wouldn’t be bad if there were more frequent dates or trainings, so that everyone had adequate training*” (*H*, P). Patients expressed high trust in HCW proficiency and correct test execution. They reported no perceived delay of processes through POC PCR-testing, positively influencing affective attitude.

### Design of standard operating procedures (SOPs) or clinical guidelines

Many stakeholders stressed the importance of standardized implementation and coordination for long-term benefits from POC PCR-testing. They highlighted the need for clear testing guidelines “*because then testing is not done all over the place, but you really have a clear guideline that says, okay, this and that must be present so that this test is justified. So that it can also achieve an optimal benefit*” (*H*, E). Restrictive SOPs were described as a means to ensure profitableness through manageable and selective use, therefore “*protecting*” (*D*, E) the workflow and economic efficiency of the healthcare unit. In settings using POC PCR, program drift, initially marked by too frequent testing without clear indications, was addressed through better adherence to testing guidelines, while some discretion in testing persisted. In tertiary hospitals, care management and hygiene experts played crucial roles in designing the SOPs. “*The guidelines are of course also set by the task force [...] and you just have to comply with them*” (*H*, P). However, some HCWs critiqued the lack of agency and involvement in designing the testing strategy: “*This is often the case […] that people who do not work here or who do not work with [the tests] make the decisions*” (*H*, P).

The scheduling of patients into cohorts in dialysis units were perceived as incompatible with the SOPs for screening patients, mainly due to low throughput of POC PCR-devices and cost.

## Sustained use of POC PCR-testing

While the feasibility of implementation presented a key prerequisite of successful long-term use, additional factors impact POC PCR-testing sustainability. Most stakeholders supported standard and extended use, noting increased staff acceptance and good patient compliance. “*I think it’s great when the test is available here and if you use it with sense and reason, you can definitely continue to use it*” (*D*, D1). Linking to workload concerns highlighted above, one long-term concern was the potential task accumulation, particularly when combined with other (future) diagnostics: “*After all, I think pandemics will always come, and if you add always more, at some point it has to be enough*” (*H*, E).

For SARS-CoV-2 testing, particularly patients voiced doubts regarding its long-term relevance and therefore efficiency due to growing public carelessness and expected reduced clinical significance of testing with less severe variants “*I don’t know what the future will bring*.” (*P*, O2). Patients generally trusted healthcare providers’ decisions: “*I assume that people know what they’re doing, and if they say now it’s necessary, then it’s okay*” (*P*, O2).

### Quality control

Decision-makers explained how the implementation and use of new diagnostics needed to comply with governmental regulations. As all study settings were in Germany, compliance with the Guidelines for laboratory tests in medical practices of the Bundesärztekammer [*Rili-BÄK*], was mandatory, and compliance with infection protection and medical product law assured quality of testing. In the tertiary hospitals, this was inter alia supervised by appointed laboratory staff. Regarding emerging quality challenges in settings using POC PCR-testing, HCWs mentioned rare occurrence of invalid results, especially when untrained staff used the devices, or forgetting to check the result of running tests in the context of daily patient care. Some noted manual documentation as a risk of data errors.

### Feedback and evaluation

In the tertiary hospital, suggestions for improvements were communicated by actively contacting superiors to give feedback, without fixed structures available. None of the interviewed HCWs had already utilized this option. They wished for more continuous participation in the discourse to ensure integrability into work processes. In low frequency testing scenarios, testing procedures were reported to “*fit well*”, with an effort within “*reasonable*” and easily manageable limits, with POC PCR-testing in the same “*handgrip*” (*D*, D1) as usual processes and examinations. HCWs reported a quick resolution of past problems with more habitual usage of tests, SOPs, appointed specialists, and manufacturer device support. Patients had either not realized they had been tested using a POC PCR-test or evaluated POC-testing related processes as efficient and well-timed, expressing high perceived effectiveness albeit rather low intervention coherence.

Appointed laboratory staff performed continuous evaluation of POC PCR-testing quality in the emergency departments. “*I was well up to date at all times about the quality of the testing on these devices and was also well able to give feedback to the task force*” (*L*1). Systematic progress monitoring (collection of meta-data) was not consistently implemented, although quality control measures were applied or considered feasible in all settings. In two study settings (P and D1), the accuracy of RSV POC PCR and the utilization of POC PCR panel tests were examined as part of a healthcare research project. Care managers were involved in evaluative discussions, but their involvement did not extend to the final decision-making process, which led to them “*proactively setting incentives*” (*D*, E) for decision-makers to consider their opinions in future testing practices.

### Intervention adaptation after implementation

The POC PCR-testing seamlessly adapted to existing workflows after implementation. *“[It has] a certain flexibility to adjust things again in the initial period as well*” (*D*, E). Devices allowed testing for different respiratory viruses or panels with different cartridges, enabling easy adaptation through continuous need-based adjustments to SOPs. In some settings, POC PCR-testing became the new standard for clinical decision-making, enhancing cost-efficiency.

To ensure sustainability, methods like technical and logistical adjustments, identification of error sources with subsequent training, and transitioning from single to panel tests with “*only one swab*” (*H*, D1) were employed. Some HCWs desired even faster diagnoses, more devices to meet testing demands, or the deployment of testing personnel in screening scenarios to uphold testing benefits.

## Discussion

In our assessment of acceptability and implementation of POC PCR-testing for respiratory infections, stakeholders described COVID-19, influenza A&B, and RSV as persisting healthcare challenges, especially in times of high prevalence. While POC PCR-tests were mostly seen as a suitable means to address these challenges in ambulatory settings frequented by vulnerable patient populations, numerous conditions hindered or facilitated successful implementation. Knowledge about POC PCR-testing was generally low, especially among non-implementors. The decision to implement POC PCR-testing depended mainly on perceived disease risk and prevalence, as well as perceived testing benefits. Decision-makers also considered governmental regulations, cost, and available funding. The process of implementation was described by most as manageable in complexity due to compatibility of POC PCR-testing with current facilities and ease of use of devices for medical personnel. Limited HCW involvement in the decision-making and planning of the implementation process resulted in some discontent. Stakeholders’ attitudes and skills were crucial for successful implementation. Most perceived the testing as improving quality of care, but some expressed opposition due to increased workload or cost concerns. Sustainability considerations highlighted the importance of standardized implementation, gradual development, adaptation to local workflows, and enforcement of SOPs.

POC PCR-device characteristics, particularly their accuracy and time-to-diagnosis, are well explored in existing literature [14,31] and were confirmed as part of quality control measures in study settings. Some previous studies have suggested a favorable impact of introducing RSV and influenza POC PCR-testing in adult patients hospitalized with respiratory illness, including reductions in microbiology test utilization [32], time-to-diagnosis, hospital stay and, thereby, in-hospital costs [33,34]. While several quantitative studies have in part explored the implementation of POC PCR-testing for SARS-CoV-2, RSV and influenza in care homes [35], emergency departments [18,36] and other clinical settings [37,38], the assessment of stakeholder perceptions on POC PCR-testing in ambulatory settings has been limited: they are often conducted through brief surveys in small cohorts or with a narrow focus on ease-of-use, thus providing only limited qualitative insights into the challenges and opportunities at both individual and systemic levels [39]. Additionally, clinical impact of COVID-19 POC PCR-testing or use of RSV/influenza/COVID-19 panel tests were not thoroughly explored at the time of study [35]. Decision-makers in our study acknowledged this lack of scientific evidence as a main barrier to POC PCR-testing implementation. This was further exacerbated by some decision-makers being unfamiliar with available literature, likely due to the limited time available beyond patient care and management [40]. This observation highlights the need for concise information on POC PCR-testing from available, trusted sources to support further scale-up of testing.

Concerning the perceived importance of the intervention, we noticed a certain paradox of patients feeling “secure” without POC PCR-testing but still supporting its implementation. This, while contradictory to the principle of tension for change as a motivational factor for complying with an intervention [22], might be explained through a notion of increased compliance with healthcare interventions in patients with a higher level of trust in their healthcare provider [41] or a lack of reflection on the current risk of transmission with passive compliance rather than active adherence [42]. This lack of information and reflection could be addressed by including communication of both test results and information about new technologies in the training of the HCWs performing the tests [43,44]. Improved patient communication has been shown to increase patient satisfaction, compliance and trust [45]. The overall level of trust in healthcare strategies in the examined groups was rather high, with critique foremost concerning feasibility of screening and only in few interviews concerning the usefulness and indication of the testing strategy itself.

The generally low awareness of scientific evidence and existing misinformation, especially among healthcare staff, merits a more pointed examination of established stakeholder information approaches. The lack of intervention coherence we observed among technically competent healthcare personnel might be explained by the high relational expense necessary to increase coherence [46], POC PCR-testing presenting only one of numerous daily tasks for healthcare professionals in our settings.

Financing implementation and sustained use posed a challenge according to stakeholders, who perceived that evidence of POC PCR’s secondary cost saving for the healthcare system was insufficient. This might impede reimbursement by healthcare insurance providers [47], and therefore aggravate already difficult cost considerations. Stakeholders described cost and financing considerations, both for POC PCRs and other novel technologies, to be one of the main barriers, especially concerning long-term use. In addition, the highlighted high cost of enabling technical integration with the hospital information system (HIS) was found to reduce the efficiency of the intervention by leading to manual documentation practices which resulted in increased workload, documentation errors, and lack of flow of information [48]. Decision-makers highlighted that isolated sponsoring or financing of initial implementation (e.g. through research funding) is a factor that has been linked to reduced capacity to maintain intervention delivery [49]. Uncertainties about necessary intervention adaptation and evolution, e.g. the changing pandemic status, waves of infection, and future population needs, present an additional challenge [50].

Cost considerations also informed stakeholders’ preferred testing strategy in our study. Targeted symptomatic or contact testing in vulnerable groups was preferred due to the cost-per-test, cumulative effort, and low throughput of POC PCR-devices. The use of POC PCR-testing in mass screening strategies was perceived as incompatible with the available budget, tense clinical workflows, and cohorted patient care. COVID-19 Ag-RDTs were perceived as more feasible in mass screening, with POC PCR-testing only for confirmation of Ag-RDT results, or before medical procedures or hospitalization. The efficiency and feasibility of this practice has previously been explored in ambulatory patient care [51] with promising results. Furthermore, stakeholders in our study acknowledged panel tests as a possible means to address the issues of task accumulation, which would increase the impact per test executed. This approach would reflect existing research where panel tests have shown comparable accuracy for included viruses as individual POC PCR-tests [52,53].

The main perceived benefit for medical personnel was facilitated patient management over prevention of transmission. Another study observed a similar effect in a primary care setting, where POC PCR-testing for SarS-CoV-2 implementation improved flow management, was described as feasible and highly accepted among patients and providers [54]. Benefits associated with facilitated decision-making and patient management were mainly reported by decision-makers, while the burden in the form of increased workload was mostly experienced by HCWs. They expressed willingness to take additional workload if it improves patient care. However, this attitude was observed to lead to discontent and decreased physical and mental wellbeing in phases of increased stress [55,56], particularly in scenarios with little agency for HCW [57]. Better communication of implementation procedures and overall benefit, as well as more involvement of HCWs in the initiative design, could therefore increase overall intervention acceptability and promote provider agency [58,59]. Given that ambulatory care settings are complex systems involving multiple interacting actors [60], the implementation of POCTs often requires a range of supporting strategies beyond structural facilitators [61]. As highlighted both in the literature [62] and our own findings, even with adequate resources in place, behavioral change among healthcare workers can prove to be difficult or delayed, underscoring the importance of addressing contextual and motivational barriers in implementation efforts. We add to this literature by identifying early involvement of end-users as well as adaptability of the intervention to fit existing workflows as important factors relating to this issue.

The conflicting balance between HCW agency and hierarchical decision-making emerged in our data, with HCW expressing they felt insufficiently considered or involved in the implementation process. The presence of a clear, functional hierarchy in hospitals, while potentially limiting healthcare worker agency, is necessary to increase efficiency and decrease workload through clear task distribution. On the other hand, if poorly executed or too strict, it can result in a dysfunctional work environment with low satisfaction of those who are subordinate [63]. As benefits of HCW involvement on implementation acceptability and success have been repeatedly highlighted [64,65], we encourage increased interprofessional exchange to promote assertiveness of HCWs and to enable identification and reporting of potential issues without recourse [66,67].

Several stakeholders in our study argued for a standardized and consistent implementation across clinical sites and the development of SOPs. Standardization is recognized to increase effectiveness of healthcare interventions, facilitate training, enable cross-coverage among staff, ensure quality of care, and prevent program drift and costly overuse, as expressed by some stakeholders [68,69]. However, standardization also reduces flexibility of adaptation to local needs or workflows. This inherent tension between standardization and customization of care delivery processes is discussed by Sinsky et al., who state a tendency towards over-standardization as a problem resulting in disempowerment and limitation of adjustments to patients’ and local circumstances [70]. This further highlights the need for exploration of patient perspectives in implementation. To achieve sustained use, we echo previous scholars highlighting the relevance of close collaboration with end-users and recipients and early consideration of issues in SOP design [71]. While implementation feasibility is a necessary first step, ongoing evaluation beyond basic quality control, including systematic monitoring and auditing of POC PCR-test use is essential for the effective and sustainable integration of these diagnostics into routine clinical practice. As highlighted in prior studies, long-term integration depends on sustained funding, maintenance of workforce skills, prioritization, and adaptation with clinical workflows [72]. In our findings, staff and patients largely supported ongoing use, though concerns emerged about cumulative workload and the diminishing relevance of SARS-CoV-2 testing. This suggests initial uptake may reflect a Hawthorne effect (an alteration of behavior by the subjects of our study due to their awareness of being observed) or novelty bias (the appearance that a new treatment or diagnostic is better when it is new), as previously observed in infection prevention research [73]. Some argue that a widespread implementation of POC PCR-testing devices and further exploration of POC PCR-testing possibilities could increase diagnostic preparedness, especially within primary healthcare [16,74]. This resonates with perceptions that some stakeholders voiced during our study.

An important factor in intervention sustainability is the persistence of clinical relevance of the tested respiratory viruses and the acknowledgement of dynamically changing health contexts requires constant intervention adaptation [75]. Annual waves of RSV and influenza, as well as persisting highly transmissible variants of SARS-CoV-2 [76], underscore their relevance for vulnerable patient collectives [77,78].

### Strengths and limitations

In our study, stakeholders had different levels of experience with POC PCR-testing and different tests were used across different study settings. This heterogeneity and size of the group led to greater variation in included experiences and thus allowed us to draw more nuanced reflections. In our sample size considerations, we aimed to strike a balance between interview-associated disruptions to clinical flows (especially for HCWs in the medical care centers) and considerations related to information sufficiency and data saturation [24] in view of our research goal [79]. We acknowledge that our stratified purposeful snowball sampling might have introduced an ascertainment bias [23]. Researcher background, relationships with participants and interview settings (COREQ [80]) are discussed in our Statement of Reflexivity (S7). The coding process was completed by one single researcher (HT), and emerging themes and codes were discussed in debriefings (IM, JW) throughout the analysis process. All interview settings were in one state (Baden-Württemberg) of one high-income country (Germany), which might merit caution when applying our conclusions to other settings, mirroring recommendations for qualitative research more generally [81].

## Conclusion

Our study highlights how POC PCR-testing implementation decisions and processes are mostly affected by knowledge about the intervention, cost, workload for HCWs, and perceived effectiveness in form of targeted patient management and prevention of transmission. Given the perceived gap in the literature regarding the clinical impact of testing, we recommend further investigation into the clinical impact of POC PCR-testing and secondary cost savings, particularly for COVID-19 and panel testing. The effort and cost of testing and the low throughput of POC PCR-devices lead to a strong tendency towards testing only upon suspicion in vulnerable patient populations, while the use in mass screening strategies is deemed unfeasible. As the burden of respiratory infections in clinical high-risk settings is expected to remain, we urge decision-makers to involve HCWs in decision-making to ensure compatibility and sustainability of strategies.

## Supporting information

S1 Fig POC PCR-testing steps. (PDF)

S2 Table Interview and Participant Characteristics. (PDF)

S3 Table Discussion Guide. (PDF)

S4 Fig The Five-Step Framework Approach. (PDF)

S5 Table Codebook Acceptability and Feasibility. (PDF)

S6 Fig Coding Trees Acceptability and Feasibility. (PDF)

S7 File Statement of Reflexivity. (PDF)

S8 Table Extended Results Acceptability Analysis. (PDF)

S9 Table Extended Results Feasibility Analysis. (PDF)

S10 File Used quotes in original language. (PDF)

S11 Table COREQ Checklist. (PDF)

## Abbreviations:

Ag-RDTs: antigen-detection Rapid Diagnostic Tests
CFIR: Consolidated Framework for Implementation Research
COVID-19: Coronavirus Disease 2019
HCW: Healthcare Worker
POC: Point-of-Care
PCR: Polymerase Chain Reaction
RSV: Respiratory Syncytial Virus
RT-PCR: Reverse Transcription Polymerase Chain Reaction
SARS-CoV-2: Severe Acute Respiratory Syndrome Coronavirus type 2
SOP: Standard Operating Procedure
TFA: Theoretical Framework of Acceptability.
