# Supplementary figures and images for: Implementation of Point-of-Care PCR-testing for the diagnosis of respiratory infections in vulnerable patient populations
Source: PLoS One. 2025 Jul 29;20(7):e0307621. doi: 10.1371/journal.pone.0307621 (PMC12306790; doi:10.1371/journal.pone.0307621)

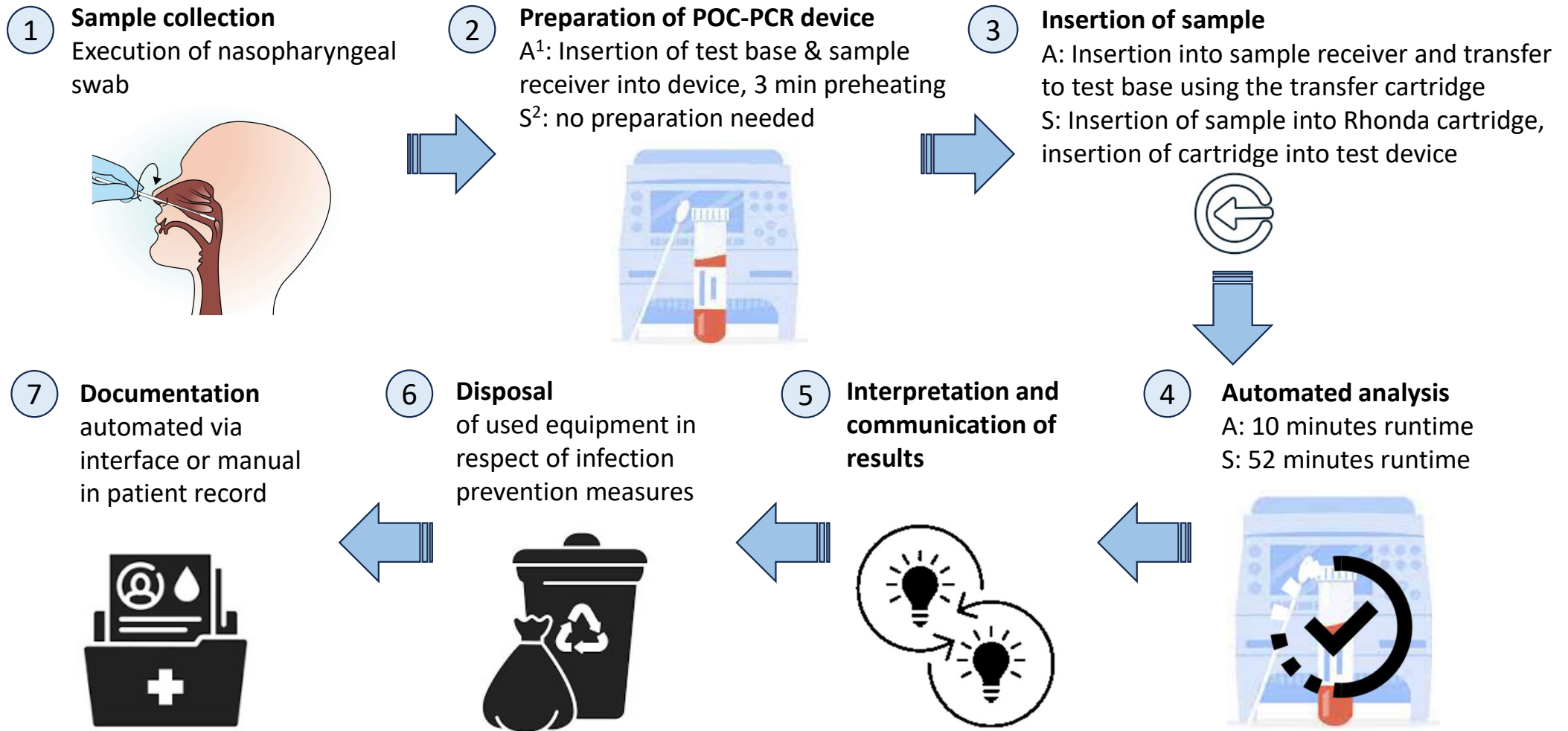

<sup>1</sup> Abbott  
<sup>2</sup> Spindiag

Supplement: S1 Fig — (PDF) [file pone.0307621.s001.pdf]
