# Supplementary material for: Implementation of Point-of-Care PCR-testing for the diagnosis of respiratory infections in vulnerable patient populations
Source: PLoS One. 2025 Jul 29;20(7):e0307621. doi: 10.1371/journal.pone.0307621 (PMC12306790; doi:10.1371/journal.pone.0307621)
Supplement: S2 Table — (PDF) [file pone.0307621.s002.pdf]

Interview number	Stakeholder group	Setting	Experience with POC PCR-testing	Test(s) used	Interview duration in minutes	Audio recording	Sex	Age of participant (age of child) in years	Years of job experience	Medical Background or Role
**1**	Patients	Pediatric Emergency Care Unit	RSV	Abbott ID Now	15	Yes	F	37 (2)	NA	Physician assistant
**2**	Patients	Pediatric Emergency Care Unit	RSV	Abbott ID Now	21	Yes	F	29 (2,5)	NA	None
**3**	Patients	Pediatric Emergency Care Unit	RSV	Abbott ID Now	15	Yes	F	30 (2)	NA	None
**4**	Patients	Pediatric Emergency Care Unit	RSV	Abbott ID Now	18	No	F	36 (3)	NA	None
**5**	Patients	Pediatric Emergency Care Unit	RSV	Abbott ID Now	17	Yes	F	31 (2)	NA	None
**6**	Patients	Ambulatory Oncology Unit (Chemo)	None	NA	14	Yes	F	67	NA	None
**7**	Patients	Ambulatory Oncology Unit (Chemo)	None	NA	14	Yes	F	73	NA	None
**8**	Patients	Ambulatory Oncology Unit (Chemo)	None	NA	20	Yes	F	38	NA	None
**9**	Patients	Ambulatory Oncology Unit (Chemo)	None	NA	17	Yes	M	59	NA	Veterinarian
**10**	Patients	Ambulatory Oncology Unit (Chemo)	None	NA	17	Yes	F	79	NA	None
**11**	Patients	Emergency Department	COVID-19	Abbott ID Now	17	Yes	M	82	NA	None
**12**	Patients	Emergency Department	COVID-19	Abbott ID Now	15	Yes	M	67	NA	None
**13**	Patients	Emergency Department, Ambulatory Oncology Unit (Chemo)	COVID-19	Abbott ID Now	23	Yes	M	56	NA	None
**14**	Patients	Emergency Department	COVID-19	Abbott ID Now	16	No	W	34	NA	None
**15**	Patients	Emergency Department	COVID-19	Abbott ID Now	14	Yes	W	91	NA	None

**16**	Patients	Dialysis Unit, Emergency Department	COVID-19	Abbott ID Now	17	Yes	W	91	NA	None
**17**	Patients	Dialysis Unit, Emergency Department	COVID-19	Abbott ID Now	15	Yes	M	78	NA	None
**18**	Patients	Dialysis Unit	COVID-19, Panel test[1]	Rhonda PCR-Schnelltestsystem (Spindiag)	14	Yes	W	58	NA	None
**19**	Patients	Dialysis Unit	COVID-19, Panel test[1]	Rhonda PCR-Schnelltestsystem (Spindiag)	13	Yes	M	72	NA	None
**20**	Patients	Dialysis Unit	COVID-19, Panel test[1]	Rhonda PCR-Schnelltestsystem (Spindiag)	18	Yes	M	74	NA	None
**21**	Healthcare workers	Pediatric Emergency Care Unit	COVID-19, RSV	Abbott ID Now	17	Yes	M	30	4	Medical resident
**22**	Healthcare workers	Pediatric Emergency Care Unit	COVID-19, RSV	Abbott ID Now	17	Yes	F	26	1	Medical resident
**23**	Healthcare workers	Pediatric Emergency Care Unit	COVID-19, RSV	Abbott ID Now	34	Yes	F	34	17	Healthcare worker
**24**	Healthcare workers	Pediatric Emergency Care Unit	COVID-19, RSV	Abbott ID Now	27	Yes	F	33	15	Healthcare worker
**25**	Healthcare workers	Ambulatory Oncology Unit (Chemo)	None	NA	18	Yes	F	39	2	Physician assistant, study representative
**26**	Healthcare workers	Emergency Department	COVID-19	Abbott ID Now	37	Yes	F	20	1	Healthcare worker
**27**	Healthcare workers	Emergency Department	COVID-19	Abbott ID Now	33	Yes	F	47	26	Healthcare worker
**28**	Healthcare workers	Emergency Department	COVID-19	Abbott ID Now	15	Yes	F	19	0	Healthcare worker

**29**	Healthcare workers	Dialysis Unit	COVID-19, Panel test[1]	Rhonda PCR-Schnelltestsystem (Spindiag)	26	Yes	F	37	16	Healthcare worker and quality management
**30**	Decisionmakers	Pediatric Emergency Care Unit	RSV	Abbott ID Now	22	Yes	M	40	11	Medical management
**31**	Decisionmakers	Pediatric Emergency Care Unit	COVID-19, RSV	Abbott ID Now	37	Yes	M	57	4	Care manager
**32**	Decisionmakers	Ambulatory Oncology Unit (Chemo)	None	NA	45	Yes	M	61	30	Medical management
**33**	Decisionmakers	Ambulatory Oncology Unit (Chemo)	None	NA	42	Yes	M	47	19	Medical management
**34**	Decisionmakers	Emergency Department	COVID-19	Abbott ID Now	32	Yes	F	37	19	Care coordinator
**35**	Decisionmakers	Emergency Department	COVID-19	Abbott ID Now	45	Yes	M	36	8	Medical management
**36**	Decisionmakers	Dialysis Unit	None	NA	33	Yes	M	53	29	Medical management
**37**	Decisionmakers	Dialysis Unit	COVID-19, Panel test[1]	Rhonda PCR-Schnelltestsystem (Spindiag)	28	Yes	M	34	13	Care manager
**38**	Decisionmakers	Dialysis Unit	COVID-19, Panel test[1]	Rhonda PCR-Schnelltestsystem (Spindiag)	16	Yes	M	39	11	Head of department
**39**	Decisionmakers	Molecular Diagnostics	COVID-19, RSV, Influenza, Panel tests	Abbott ID Now, Roche	52	Yes	M	54	30	Head of molecular diagnostics POC-representative
**40**	Decisionmakers	Molecular Diagnostics	COVID-19, RSV, Influenza, Panel tests	Abbott ID Now, Cobas (Roche), NS[2]	45	Yes	M	50	27	Head of molecular diagnostics

1 Panel test of COVID-19, Influenza A&B and RSV

2 not specified
