# Supplementary material for: Implementation of Point-of-Care PCR-testing for the diagnosis of respiratory infections in vulnerable patient populations
Source: PLoS One. 2025 Jul 29;20(7):e0307621. doi: 10.1371/journal.pone.0307621 (PMC12306790; doi:10.1371/journal.pone.0307621)
Supplement: S3 Table — (PDF) [file pone.0307621.s003.pdf]

Themenleitfaden für die Befragung von Interessengruppen:

Research Questions

1. *What is the acceptability of SARS-CoV-2 and influenza POC PCR testing among stakeholders, including patients (or, in the pediatric emergency care unit, the legal guardian), healthcare workers, and decision makers?*
2. *Is POC PCR testing a feasible method for the early detection of SARS-CoV-2 and influenza infections in ambulatory care settings attended by vulnerable patient populations?*
3. *What would be the requirements for a scale-up of POC PCR testing?*

Übersicht Theoretical Frameworks

1. Akzeptanz (in Anlehnung an Sekhan et al. Acceptability Theoretical Framework for Healthcare Interventions)

1. ***Affective Attitude*** *(how an individual feels about the intervention)*
2. ***Burden*** *(the perceived amount of effort that is required to participate in the intervention)*
3. ***Ethicality*** *(the extent to which the intervention has good fit with an individual's value system)*
4. ***Intervention Coherence*** *(the extent to which a participant understands the intervention and how it works)*
5. ***Opportunity Costs*** *(the extent to which benefits, profits, values have to be given up to engage in the intervention)*
6. ***Perceived Effectiveness*** *(the extent to which the intervention is perceived as likely to achieve its purpose)*
7. ***Self-efficacy*** *(the participant's confidence that they can perform the behaviors required to participate in the interventions)*

2. Durchführbarkeit (in Anlehnung an Consolidated Framework for Implementation Research)

3. Anforderungen für Scale-Up (nur für medizinisches Fachpersonal und Entscheidungsträger)

Nützliche Probes:

Was meinen Sie mit...?

Wie war die Situation?

Wie haben Sie sich dabei gefühlt?

Was haben Sie darüber gedacht?

Wie war das für Sie?

Erklären Sie bitte, was / wie sie das Sie meinen.

Erzählen Sie mir bitte mehr darüber.

Verwendung von Schweigen, mhm, uh huh, oder 1-Wort Wiederholungen, um weitere Ausführungen zu fördern.

Können Sie ein Beispiel nennen?

Wie haben es andere gesehen?

Welche Art von Feedback haben Sie erhalten?

Was hat Ihr Vorgesetzter gesagt/getan?

Ich bin nicht sicher, ob ich verstehe, was Sie meinen.

Vorhin haben Sie erwähnt, dass X. Könnte das ein Beispiel für Y sein?

Wie verhält sich das zu dem, was Sie gerade meinten?

Warum? vermeiden

Themenleitfaden für die Befragung von Interessengruppen:

Medizinisches Fachpersonal

Dieser Themenleitfaden beschreibt die Bereiche, die in den qualitativen Interviews behandelt werden sollen. Der Interviewer kann während des Gesprächs zusätzliche Fragen stellen, um Informationen zu klären. Die Fragen können während der Befragung geringfügig angepasst werden, wenn Beobachtungen oder alternative Fragen entstehen.

1. Einführungen

Erläuterung von Ethik, Einwilligung und Vertraulichkeit der Befragung und Analyse.

Erklärung für die Teilnehmer, dass es um Point-of-Care PCR-Tests für Atemwegserkrankungen wie COVID-19, Influenza und RSV in ambulanten Einrichtungen mit Hochrisikopatienten, sowie deren Durchführung geht. Betonung, warum wir uns für ihre Ansichten interessieren.

Erläuterung des Ablaufs und der Dauer des Interviews.

Haben Sie Fragen?

Einschalten des Aufnahmegeräts, nachdem das Informationsblatt und die Einwilligungserklärung unterschrieben wurden.

*Achten Sie darauf, dass Sie die folgenden Informationen sammeln und zu Beginn des Gesprächs in den **Zusammenfassungsbogen** eintragen:*

Teilnehmercode:

Geschlecht:

Stakeholder-Gruppe:

Personal: Jahre Berufserfahrung

Zeitpunkt des Interviews:

Ort des Interviews:

Name des Interviewers:

Kontext: Bitte beschreiben Sie kurz Ihr Aufgabenspektrum und Ihre Position in Ihrer Einrichtung.

Was verstehen Sie unter POC PCR-Tests? / Welche Rolle spielen POC PCR-Tests in Ihrer Einrichtung?

2. Themen des Interviews

1: Erfahrungen und Testablauf

Wie waren Ihre bisherigen Erfahrungen mit der aktuellen Teststrategie zur Diagnostik von respiratorischen Erkrankungen wie COVID-19, RSV oder Influenza?

a) Welche Tests genau werden verwendet?

Probes: Könnten Sie kurz den Ablauf der Testung in Ihrer Einrichtung zusammenfassen? Wie lange verwenden Sie diese Teststrategie bereits? Welche Konsequenz hat das Ergebnis?

b) War die mögliche Verwendung von POC PCR-Test in Ihrer Einrichtung bereits ein Thema?

Probes: Haben Sie die Absicht, in der Zukunft POC PCR-Tests zu verwenden?

c) *Wenn ja:* Was sind Ihre Erwartungen an die neue Einführung von POC PCR-Tests zur Diagnostik von Atemwegserkrankungen?
Wenn nein: Was spricht für Sie gegen eine Einführung?

d) Hätte die Verwendung von POC PCR Tests einen Einfluss auf Ihre Arbeitsbelastung?

Probes: Nutzerfreundlichkeit der Geräte? Handhabung? Zeitaufwand?

e) Was wären in Ihren Augen die Vorteile von POC-PCRs?

Probes: Haben Sie das Gefühl, dass Sie / Ihre Patienten von diesen Teststrategien profitieren würden? Wenn ja, wie? Fällt Ihnen ein konkretes Beispiel ein?

f) Welche Probleme oder Hindernisse könnten auftreten?

Probes: Als wie relevant für die praktische Durchführung von POC PCR-Tests empfinden Sie diese?

2: Wahrnehmung der Intervention

Wie wäre die allgemeine Reaktion des Gesundheitspersonals und der Patienten auf die Einführung von POC PCR-Tests?

a) Wie nehmen Sie die Einstellung der Zentrallabore und deren Mitarbeiter zu POC-PCR-Tests wahr?

b) Warum könnten Menschen die Einführung / Verwendung von Point-of-Care-PCR-Diagnostik als problematisch wahrnehmen?

Probes: Teilen Sie diese Bedenken?

War das in Ihrer eigenen Einrichtung auch ein Thema? (*nur wenn bereits über POC PCR gesprochen wurde)*

3: Einschätzung der Effektivität

Welchen Einfluss hat das Einführen von POC PCR-Diagnostik auf die Verbreitung und die Kontrolle von respiratorischen Infektionen?

a) Wie sind Sie zu dieser Einschätzung gekommen?

Probes: Persönliche Erfahrung? Wissenschaftliche Literatur? Konferenzen? Kollegen?

b) Für wie zuverlässig halten Sie die Testergebnisse?

Probes: falsch positiv, falsch negativ?

c) Glauben Sie, dass diese Auswirkungen den Aufwand der Einführung von POC PCR-Diagnostik rechtfertigen?

d) Wie würden Sie die Kosten für die Einführung und laufende Kosten einschätzen?

4: Ablauf des Einführungsprozesses

Könnten Sie mir beschreiben, wie Sie sich den Einführungsprozess der Testverfahren in Ihren klinischen Arbeitsalltag idealerweise vorstellen würden?

a) Wer sollte die Entscheidung treffen?

b) Wie sollte Personal im Umgang mit den Geräten geschult werden?

Probes: Ablauf der Schulung? Wer sollte eine Schulung erhalten? Wie häufig?

c) Wie stark sollten Sie als medizinische Fachangestellte in den Einführungsprozess eingebunden werden?

Probes: Planung? Nur die Möglichkeit Feedback zu geben?

d) Welche logistischen Änderungen wären für die Einführung von POC PCR-Tests bei Ihnen in der Ambulanz nötig?

Probes: Lagerung, Probenverarbeitung, Entsorgung?

5: Scale Up

Was wäre nötig, um die POC-PCR-Diagnostik für respiratorische Infekte in allen ambulanten onkologischen Abteilungen oder Dialyseeinheiten in Deutschland erfolgreich einzuführen?

Probes: Kosten und Finanzierung? Guidelines? Abrechnung?

a) Welche Maßnahmen sind Ihrer Meinung nach zur Qualitätssicherung notwendig?

Probes: Personalschulung, häufige Qualitätskontrollen, Unterstützung durch Labore?

b) Würden Sie einen dauerhaften Einsatz von POC PCR-Tests für Atemwegsinfektionen in Ihrer Einrichtung befürworten?

Probes: Wie kommen Sie zu dieser Einschätzung?

c) Was sollte bei so einem Scale-up vermieden werden?

3. Abschluss

Gibt es noch etwas, das Sie mir gerne mitteilen möchten? / Haben wir etwas noch nicht besprochen, das Sie als wichtig erachten? Wenn Ihnen noch etwas einfällt steht meine Kontaktadresse auch auf der Informationsschrift.

Dem Probanden für seine Teilnahme danken.

Themenleitfaden für die Befragung von Interessenvertretern:

Patienten / gesetzliche Vertreter

Dieser Themenleitfaden beschreibt die Bereiche, die in den qualitativen Interviews behandelt werden sollen. Der Interviewer kann während des Gesprächs zusätzliche Fragen stellen, um Informationen zu klären. Die Fragen können während der Befragung geringfügig angepasst werden, wenn Beobachtungen oder alternative Fragen entstehen.

1. Einführungen

Erläuterung von Ethik, Einwilligung und Vertraulichkeit der Befragung und Analyse.

Erklärung für die Teilnehmer, dass es um Point-of-Care PCR-Tests für Atemwegserkrankungen wie COVID-19, Influenza und RSV in ambulanten Einrichtungen mit Hochrisikopatienten, sowie deren Durchführung geht. Betonung, warum wir uns für ihre Ansichten interessieren.

Erläuterung des Ablaufs und der Dauer des Interviews.

Haben Sie Fragen?

Einschalten des Aufnahmegeräts, nachdem das Informationsblatt und die Einwilligungserklärung unterschrieben wurden.

*Achten Sie darauf, dass Sie die folgenden Informationen sammeln und zu Beginn des Gesprächs in den **Zusammenfassungsbogen** eintragen:*

Teilnehmercode:

Geschlecht:

Stakeholder-Gruppe:

Patienten: höchster Bildungsabschluss, Alter

Zeitpunkt des Interviews:

Ort des Interviews:

Name des Interviewers:

Kontext: Wurden Sie bereits mit einem POC-PCR Test getestet?

Sind Sie zur ambulanten Behandlung oder für eine stationäre Aufnahme gekommen?

Als wie gefährdet sehen Sie sich in Bezug auf Ansteckung oder einen schweren Verlauf bei COVID-19 und andere Atemwegsinfektionen wie Grippe?

2. Themen des Interviews

1: Erfahrungen und Testablauf (kurz)

Welche Erfahrungen haben Sie bisher mit COVID- und Grippe-Diagnostik in der Ambulanz bzw für Ihren Besuch in der Ambulanz gemacht?

a) Können Sie mir kurz zusammenfassen, wie das Testen selbst abgelaufen ist?

    Probes: Wissen Sie, welche Tests verwendet wurden / wofür die Tests waren? Wie wurden Sie abgestrichen / Ihnen das Ergebnis mitgeteilt?

b) Wie gefällt Ihnen der aktuelle Ablauf des Testens für COVID-19 oder Influenza?
c) Sind im Zusammenhang mit dem Testen Probleme aufgetreten?
d) Gibt es etwas, das Sie am aktuellen Vorgehen verändern würden?

Wie würde Ihnen die Einführung der Testung mit COVID, RSV oder Infuenza PCR Tests direkt in der Ambulanz gefallen?

2: Kompatibilität der Einführung mit Klinikalltag

Können Sie sich vorstellen, wie sich der Ablauf Ihres Besuchs in der Ambulanz verändern würde, wenn Sie statt der aktuellen Teststrategie mit POC PCR-Test getestet werden würden?

a) Hätte es einen Einfluss auf den Zeitaufwand Ihres Ambulanzbesuchs?
b) Wie würde sich die Wartezeit in der Ambulanz verändern?
c) Würden Sie sich sicherer / weniger sicher fühlen?
d) Welchen Einfluss hätte das geänderte Testen auf Ihre Aufnahme/Behandlung?

3: Wahrnehmung der Intervention

Wie wäre Ihre Reaktion auf die Einführung von Point-of-Care PCR-Tests in der Ambulanz?

a) Was wären Ihre Erwartungen, wenn Ihnen ein POC PCR-Test zur Diagnostik von Atemwegserkrankungen vor Ort angeboten würde?
b) Warum könnten Menschen die Einführung / Verwendung von PCR-Diagnostik am Ort der Patientenversorgung als problematisch wahrnehmen?

    Probes: Teilen Sie diese Bedenken?

c) Was sind Ihrer Meinung nach die Vor- und Nachteile der Verwendung von PCR-Tests in der Diagnostik am Ort der Patientenversorgung?

    Probes: Haben Sie das Gefühl, dass Sie von diesen Teststrategien profitieren würden?

d) Fällt Ihnen ein konkretes Beispiel ein?

4: Einschätzung der Effektivität

Glauben Sie, dass POC PCR-Tests einen Einfluss auf die Verbreitung und die Kontrolle von Infektionen haben können?

a) Wie sind Sie zu dieser Einschätzung gekommen?

Probes: Persönliche Erfahrung? Berichterstattung? Bekannte?

b) Wie sehr vertrauen Sie den Testergebnissen?

5: Scale Up

Halten Sie es für sinnvoll, PCR-Screenings auf Atemwegsinfektionen am Ort der Patientenversorgung in mehr Kliniken einzuführen?

a) Wie könnte sich das auf Ihre zukünftigen Klinik- oder Arztbesuche auswirken?
b) Wann (unter welchen Umständen) sollte Ihnen ein solcher Test angeboten werden?
c) Würden Sie es vorziehen, sich außerhalb der Klinik testen zu lassen, bevor Sie zu Ihrem Termin kommen?

3. Abschluss

Gibt es noch etwas, das Sie mir gerne mitteilen möchten? / Haben wir etwas noch nicht besprochen, das Sie als wichtig erachten?

Dem Probanden für seine Teilnahme danken.

Themenleitfaden für die Befragung von Interessengruppen: Entscheidungsträger

Dieser Themenleitfaden beschreibt die Bereiche, die in den qualitativen Interviews behandelt werden sollen. Der Interviewer kann während des Gesprächs zusätzliche Fragen stellen, um Informationen zu klären. Die Fragen können während der Befragung geringfügig angepasst werden, wenn Beobachtungen oder alternative Fragen entstehen.

1. Einführungen

Erläuterung von Ethik, Einwilligung und Vertraulichkeit der Befragung und Analyse.

Erklärung für die Teilnehmer, dass es um Point-of-Care PCR-Tests für Atemwegserkrankungen wie COVID-19, Influenza und RSV in ambulanten Einrichtungen mit Hochrisikopatienten, sowie deren Durchführung geht. Betonung, warum wir uns für ihre Ansichten interessieren.

Erläuterung des Ablaufs und der Dauer des Interviews.

Haben Sie Fragen?

Einschalten des Aufnahmegeräts, nachdem das Informationsblatt und die Einwilligungserklärung unterschrieben wurden.

*Achten Sie darauf, dass Sie die folgenden Informationen sammeln und zu Beginn des Gesprächs in den **Zusammenfassungsbogen** eintragen:*

Teilnehmercode:

Geschlecht:

Stakeholder-Gruppe:

Personal: Jahre Berufserfahrung

Zeitpunkt des Interviews:

Ort des Interviews:

Name des Interviewers:

Kontext: Bitte beschreiben Sie kurz Ihre Rolle und Position in Ihrer Einrichtung.

Was sind Ihre spezifischen Aufgaben und Verantwortlichkeiten in Bezug auf Diagnostik respiratorischer Atemwegsinfekte in Ihrer Einrichtung?

2. Themen der Befragung

1: Erfahrungen und Testablauf

Welche Erfahrungen haben Sie bisher mit verschiedenen Teststrategien zur Diagnostik von Atemwegsinfekten gemacht?

a) Was ist das aktuelle Vorgehen bezüglich der Diagnostik respiratorischer Infektionen in Ihrer Abteilung?

   Probes: Welche Tests werden verwendet? Wie lange verwenden Sie in Ihrer Einrichtung bereits die aktuelle Teststrategie? Wo werden die Tests durchgeführt? Wer wird getestet? Von wem werden die Tests durchgeführt? Wie werden positive Ergebnisse gehandhabt?

b) Wie würden Sie die Einführung von POC PCR-Tests zur Diagnostik von Atemwegserkrankungen bewerten?

   Probes: Wie bewerten Sie POC PCR-Tests im Vergleich zum Testen mit RT-PCR im Labor?

c) Was sind Ihrer Meinung nach die Vorteile der Verwendung von POC-PCR?

   Probes: Haben Sie das Gefühl, dass Ihre Einrichtung / Ihre Patienten von diesen Teststrategien profitieren würde?

d) Welche Probleme könnten in Zusammenhang mit der Anwendung von POC PCR-Tests auftreten?

   Probes: Wie könnten diese überwunden werden?

e) Welche Befürchtungen haben Sie hinsichtlich der Durchführbarkeit? Was erhoffen Sie sich?
f) Gibt es etwas, das Sie der Teststrategie hinzufügen oder an ihr ändern würden?

2: Wahrnehmung der Intervention

Wie schätzen Sie die allgemeine Reaktion des Gesundheitspersonals und der Patienten auf die Einführung von POC PCR-Tests ein?

   Probes: Haben Sie bereits Rückmeldungen oder Anregungen von Mitarbeitern oder Patienten bezüglich der Teststrategie erhalten?

a) Wie würden Sie die Einstellung der Zentrallaboratorien und deren Mitarbeiter zu POC-PCR-Tests einschätzen?
b) Warum könnten Menschen die Einführung / Verwendung von Point-of-Care-PCR-Diagnostik als problematisch wahrnehmen?

   Probes: Teilen Sie diese Bedenken?

c) Wie beurteilen Sie persönlich das Potenzial von POC-PCR-Tests für das Screening von Atemwegsinfektionen?

3: Ablauf des Einführungsprozesses

Wie würde der ideale Prozess der Einführung von POC PCR-Tests in Ihrer Einrichtung aussehen?

a) Wer sollte an der Entscheidungsfindung während der Implementierungsstrategie beteiligt sein?
b) *Nur wenn Intention:* Warum haben Sie als Institution bisher keine POC PCR-Diagnostik für Atemwegsinfekte eingeführt?
c) Wie sollte die Einführung an das Personal kommuniziert werden? Wie sollte das Personal im Umgang mit den Tests geschult werden?
d) Welche Faktoren könnten die Umsetzung der neuen Teststrategie erleichtern?
e) Welche Schwierigkeiten/Hindernisse könnte es bei der Einführung der neuen POC PCR-Diagnostik in Ihrer Einrichtung geben?

Probes: Logistik? Datenmanagement? Kosten?

4: Einschätzung der Effektivität

Welchen Einfluss hätte das Einführen von POC PCR-Diagnostik auf die Verbreitung und die Kontrolle von respiratorischen Infektionen?

a) Wie sind Sie zu dieser Einschätzung gekommen?

Probes: Persönliche Erfahrung? Wissenschaftliche Literatur? Konferenzen? Kollegen?

b) Für wie zuverlässig halten Sie die Testergebnisse?

Probes: Falsch positiv, falsch negativ?

c) Glauben Sie, dass diese Auswirkungen den Aufwand der Einführung von POC PCR-Diagnostik in Ihrer Abteilung rechtfertigen würden?

5: Scale Up

Was wäre nötig, um die POC-PCR-Diagnostik für respiratorische Infekte in allen ambulanten onkologischen Abteilungen oder Dialyseeinheiten in Deutschland erfolgreich einzuführen?

Probes: Anreize? Kosten und Finanzierung? Guidelines?

a) Wer sollte involviert werden?
b) Welche Maßnahmen sind Ihrer Meinung nach zur Qualitätssicherung notwendig?

Probes: Personalschulung? häufige Qualitätskontrollen? Unterstützung durch Labore?

c) Was sollte vermieden werden?
d) Würden Sie einen dauerhaften Einsatz von POC PCR-Tests für Atemwegsinfektionen in Ihrer Einrichtung befürworten?

Probes: Wie kommen Sie zu dieser Einschätzung?

e) Was hält Ihrer Meinung nach andere Entscheidungsträger davon ab, POC-PCR-Tests für die Diagnostik von Atemwegserkrankungen in ihrer Einrichtung einzuführen?

Probes: Wie könnten diese Probleme angegangen werden?

3. Abschluss

Gibt es noch etwas, das Sie mir gerne mitteilen möchten? / Haben wir etwas noch nicht besprochen, dass Sie als wichtig erachten?

Dem Probanden für seine Teilnahme danken.
