# Supplementary material for: Implementation of Point-of-Care PCR-testing for the diagnosis of respiratory infections in vulnerable patient populations
Source: PLoS One. 2025 Jul 29;20(7):e0307621. doi: 10.1371/journal.pone.0307621 (PMC12306790; doi:10.1371/journal.pone.0307621)
Supplement: S4 Fig — (PDF) [file pone.0307621.s004.pdf]

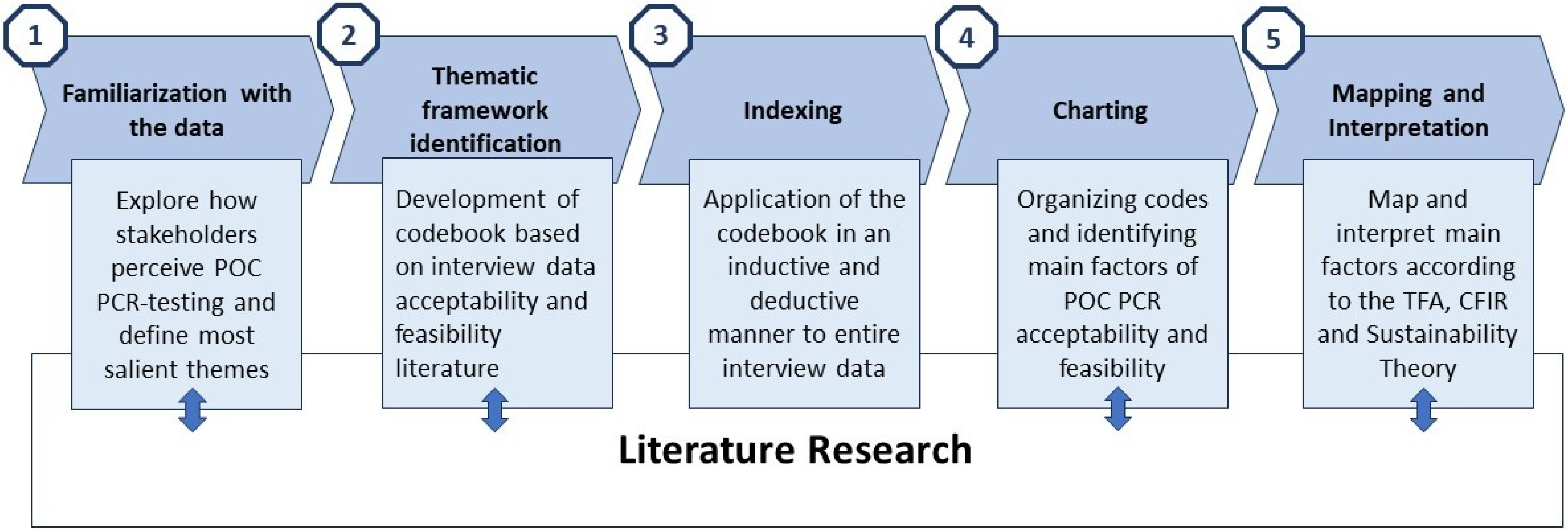

POC: Point-of-Care
TFA: Thematic Framework of Acceptability
CFIR: Consolidated Framework of Implementation Research
