# Supplementary material for: Implementation of Point-of-Care PCR-testing for the diagnosis of respiratory infections in vulnerable patient populations
Source: PLoS One. 2025 Jul 29;20(7):e0307621. doi: 10.1371/journal.pone.0307621 (PMC12306790; doi:10.1371/journal.pone.0307621)
Supplement: S5 Table — (PDF) [file pone.0307621.s005.pdf]

CODEBOOKS

Acceptability of POC PCR-testing

Anticipated (forward-looking) = attitude before actual usage of POC PCR-testing or actual experience of the stated consequence. *Include statements from interviewees without experience in POC PCR-testing and retrospective statements about attitudes and perceptions before the implementation.*

Concurrent (backward-looking) = attitude towards POC PCR-testing during or right after actual usage or having experienced the stated consequence. *Include statements from interviewees with experience in POC PCR-testing.*

Code	Definition	Number of interviews	Number of text units	Temporal perspective
**Affective Attitude**	How an individual feels about POC PCR-testing for respiratory infections. This includes statements related to emotional evaluation of the intervention and its consequences.			
Compliance	The extend to which the stakeholders' behaviour coincides with the intentions of the POC PCR-testing strategy. *Include statements that refer to compliance of involved individuals with the intervention.*	8	12	Anticipated
		15	25	Concurrent
Conditionality	The approval of POC PCR-testing under specific conditions. *Include statements that reflect under which conditions POC PCR-testing is perceived as effective, helpful or necessary.*	19	62	Anticipated
		22	89	Concurrent
Feeling of security	The perceived safety of stakeholders in the institution concerning respiratory viral infections. *Include statements that show how POC PCR-testing influences an individual's feeling of security while visiting the institution.*	7	18	Anticipated
		17	23	Concurrent
Cause of insecurities		2	3	Anticipated

Code	Definition	Number of interviews	Number of text units	Temporal perspective
	The perception of POC PCR-testing as cause of uncertainty or anxiety. *Include statements referring to POC PCR-testing and the testing strategy as a cause of insecurity.*	0	0	Concurrent
Pandemic fatigue	The emerging demotivation to engage in protection behaviours and seek COVID-19-related information due to complacency, alienation and hopelessness. *Include statements that show a notion of pandemic fatigue that impacts the view of POC PCR-testing.*	6	7	Anticipated
		3	3	Concurrent
Passivity	The acceptance of POC PCR-testing, without active response or resistance. *Include statements that show an inactivity, indifference or passive compliance with POC PCR-testing.*	3	3	Anticipated
		4	6	Concurrent
Perceived benefit	The positive or advantageous effect of POC PCR-testing for the individual or the institution that is observed or expected. *Include statements that refer to the perceived benefit of the intervention, when participants express these aspects with an affective connotation or evaluation.*	9	23	Anticipated
		16	44	Concurrent
Perceived importance	The importance the stakeholders assign to POC PCR-testing in the diagnostic strategy and care management at the institution. *Include statements that reflect the perceived importance of POC PCR-testing or its implementation in the given context.*	24	78	Anticipated
		29	152	Concurrent
Perceived risk	The stakeholders' subjective evaluation of their risk of an adverse outcome concerning transmission of respiratory viral infections or course of disease. *Include statements that refer to the perceived risk of infection or severe course of disease of the patient or staff collective at the given setting.*	13	50	Anticipated
		22	77	Concurrent

Code	Definition	Number of interviews	Number of text units	Temporal perspective
Reluctance	The unwillingness or disinclination to participate in POC PCR-testing. *Include statements that show a notion of disapproval or reluctance towards the intervention.*	13	50	Anticipated
		18	42	Concurrent
Satisfaction	The level of enjoyment and reward stakeholders and staff get from participating in the initiative. *Include statements about how satisfied the participants are with the intervention.*	13	28	Anticipated
		25	76	Concurrent
Scepticism	A questioning attitude or doubt toward the usage or appropriateness of POC PCR-testing. *Include all statements that show a feeling of scepticism towards the intervention.*	5	6	Anticipated
		7	9	Concurrent
Fear of faulty use	The wrong handling of POC PCR-testing devices and testing procedures. *Include statements that depict a fear of faulty use of the intervention including wrong execution and non-compliance with quality standards.*	8	16	Anticipated
		7	15	Concurrent
Fear of misuse	The use of POC PCR in an improper way or for wrong purposes. *Include statements that reflect a fear of misuse in the sense of wrong indication and overuse of the intervention.*	2	6	Anticipated
		1	1	Concurrent
Sense of incapacitation	The feeling of being deprived of power or autonomy due to POC PCR-testing. *Include statements that show that patients feel incapacitated due to POC PCR-testing and associated strategies*.	3	5	Anticipated
		0	0	Concurrent
Trust	The belief that POC PCR-testing and the chosen strategy are safe and reliable. *Include statements that refer to a sense of trust in the intervention and a feeling of trust in the right choice of testing strategy by the institution.*	1	1	Anticipated
		24	55	Concurrent

Code	Definition	Number of interviews	Number of text units	Temporal perspective
Correct execution	The correct performance of procedures necessary for carrying out POC PCR-testing. *Include statements that show the participants trust in correct execution of the intervention including swabs and device handling.*	3	4	Anticipated
		6	7	Concurrent
**Burden**	The perceived amount of effort that is required to participate in POC PCR-testing including time, expense, or cognitive effort.			
Access	The opportunity or right to obtain or use POC PCR-testing. *Include statements referring to limited or restricted access to POCT technology or the needed materials to run the intervention.*	2	3	Anticipated
		7	10	Concurrent
Consequences of result	The unwelcome or unpleasant results of POC PCR-testing. *Include statements about burden evolving from the consequences of the result such as needed hygiene measures or rescheduling of appointments.*	6	8	Anticipated
		23	44	Concurrent
Stigma through positive result	Society's negative evaluation of someone testing positive for COVID-19. *Include statements about experienced or feared stigma due to positive COVID status revealed through POC PCR-testing.*	0	0	Anticipated
		1	1	Concurrent
Cost	The price paid to acquire and maintain POC PCR-testing. *Include statements related to financial cost of the intervention.*	11	32	Anticipated
		15	44	Concurrent
Reimbursement	The payment of the expenses incurred as a loss covered by an insurance policy. *Include statements referring to reimbursement of money paid for the intervention and possible difficulties associated with reimbursement.*	3	12	Anticipated
		4	5	Concurrent

Code	Definition	Number of interviews	Number of text units	Temporal perspective
Staff	The required set of people who make up the workforce necessary for performing POC PCR-testing. *Include statements referring to needed human resources in order to participate in the intervention.*	1	4	Anticipated
		1	1	Concurrent
Data management	The process of ingesting, storing, organizing and maintaining the data created and collected. *Include statements referring to the burden of data management such as data entry into the testing device, transfer of results and management of the hospital information system.*	3	7	Anticipated
		12	30	Concurrent
Discomfort	The feeling of being uncomfortable physically or mentally caused by POC-PCR-testing. *Include statements referring to perceived discomfort caused by the intervention such as nasal swab or discomfort due to testing accommodation.*	7	13	Anticipated
		14	24	Concurrent
Disruption of processes	An interruption in the normal course or continuation of clinical processes. *Include statements referring to a disruption of normal work processes on an institutional level due to POC PCR-testing; code to "workflow" if this affects the individual.*	5	18	Anticipated
		13	18	Concurrent
Logistics	The overall process of managing how resources are acquired, stored, and transported. *Include statements about burden of POC PCR-testing through necessary logistics such as room, transports, waste disposal and orderings.*	6	22	Anticipated
		17	54	Concurrent
Waste	Unwanted or unusable material, substances, or by-products of POC PCR-testing. *Include statements referring to waste that is generated through the intervention.*	0	0	Anticipated
		3	5	Concurrent

Code	Definition	Number of interviews	Number of text units	Temporal perspective
Low turnover	The number of possible performed tests per time unit. *Add statements referring to the low turnover of POC PCR devices and its consequences.*	1	1	Anticipated
		7	8	Concurrent
No quantification	The possibility to quantify the amount of viral DNA per sample unit, e.g. the number of cycles required for the fluorescent signal to cross the threshold. *Include statements referring to the lack of quantification in POC PCR-testing and its consequences.*	0	0	Anticipated
		5	7	Concurrent
Overuse	Excessive use of POC PCR-testing upon availability. *Include statements referring to the burden through overuse of the intervention; double code with "Fear of Misuse" if emotional component.*	2	2	Anticipated
		5	6	Concurrent
Quality control	The set of procedures intended to ensure that a performed service adheres to a defined set of quality criteria. *Include statements on perceived burden through necessary measures for quality control such as staff training or ring testing.*	4	9	Anticipated
		8	19	Concurrent
Transmission through contact	The transfer of an infection from person to person through physical proximity. *Include all statements referring to exposure and risk of transmission due to on-site testing.*	3	5	Anticipated
		0	0	Concurrent
Waiting time	The amount of time the patients or staff must wait before continuing care processes. *Include statements on waiting time due to POC PCR-testing for patients waiting for the result or staff when using the device or when waiting for the device to be free.*	9	13	Anticipated
		23	46	Concurrent

Code	Definition	Number of interviews	Number of text units	Temporal perspective
Workload	The added effort and amount of work to be done by a particular person in a period of time when POC PCR-testing is implemented. *Include statements related to changes in workload due to the intervention and statements about additional time requirements.*	10	28	Anticipated
		18	75	Concurrent
**Ethicality**	The extent to which POC PCR-testing has a good fit with an individual's value system. This includes how rational individuals perceive the intervention and how they would ideally use or implement it.			
Altruism	Unselfish actions concerned with the well-being of others *Include statements that reveal a motivation to participate in POC PCR-testing even at the individual's own disadvantage out of concern for others or the greater good.*	3	7	Anticipated
		6	6	Concurrent
Implicitness	The degree to wich the use of POC PCR-testing is perceived as naturally implied or unquestioned by stakeholders. *Include statements that show a notion of implicitness towards the intervention and where participants show a sense of naturalness of the intervention.*	4	7	Anticipated
		19	27	Concurrent
Involvement	The degree to which stakeholders actively take part in the intervention. *Include statements that show the degree of personal and emotional involvement in POC PCR-testing of the participants.*	3	4	Anticipated
		9	18	Concurrent
Responsibility	The moral duty to do something because of your job, position, or values. *Include statements that show a sense of responsibility as a motivator to participate in POC PCR-testing.*	5	6	Anticipated
		11	14	Concurrent
Protection of others		6	11	Anticipated

Code	Definition	Number of interviews	Number of text units	Temporal perspective
	Keeping others safe from injury, damage, or loss through participating in the intervention. *Include statements that show the wish to protect others as a motive behind participation in POC PCR-testing. Code all statements that include a sort of disadvantage for oneself to "Altruism".*	16	20	Concurrent
Sense of obligation	The notion of being morally or legally bound to participating in an intervention. *Include statements that show that the participant participates in POC PCR-testing out of a sense of obligation either towards their institution or the society.*	5	6	Anticipated
		7	8	Concurrent
**Intervention Coherence**	The extent to which a participant understands POC PCR-testing, its purpose and how it works.			
Insecurity	The lack of confidence in one's own knowledge about the intervention. *Include all statements that refer to stakeholders' insecurity regarding knowledge of the POC PCR-testing and strategies.*	9	14	Anticipated
		22	41	Concurrent
Knowledge	The theoretical or practical understanding of an intervention. *Include statements that show the understanding of POC PCR-testing and how it works in relation to the problem it targets as well as statements referring to required knowledge associated with the testing.*	8	35	Anticipated
		29	111	Concurrent
Misperception	The wrong or incorrect understanding or interpretation of an intervention. *Include all statements that reveal that the participant has misperceptions about POC PCR-testing as well as obvious contradictions with other participant's statements.*	4	6	Anticipated
		17	31	Concurrent
**Opportunity Costs**	The extent to which benefits, profits, values have to be given up to engage in POC PCR-testing.			

Code	Definition	Number of interviews	Number of text units	Temporal perspective
Interference	The negative alteration or disruption of other important processes. Include *statements referring to interference of the POC PCR-testing strategy with other heath related practices.*	2	2	Anticipated
		7	14	Concurrent
Alternative strategies	Other possible strategies to manage respiratory viral infections. *Include statements related to the benefit, perceived usefulness or sufficiency of other testing practices and protective measures that are not POC PCR-testing.*	9	26	Anticipated
		22	59	Concurrent
**Perceived Effectiveness**	The extent to which POC PCR-testing is perceived as likely to achieve its purpose.			
Accuracy	The degree of closeness of measurements to the analyte's true value. *Include all statements concerning sensitivity, specificity, and perceived trustworthiness of results.*	9	14	Anticipated
		24	59	Concurrent
Availability of testing	The quality of being close at hand and ready for use. *Include all statements referring to availability of testing opportunities through POC PCR-testing or constraints of testing due to alternatives.*	1	1	Anticipated
		5	7	Concurrent
Contribution to diagnostic process	The part played by an intervention in bringing about a result that affects the clinical reasoning. *Include all statements referring to how POC PCR-testing and the knowledge of the patient's infection status influence the diagnostic process.*	5	6	Anticipated
		18	38	Concurrent
Patient comfort	The patient being in the condition of well-being, contentment, and security. *Include statements referring to how the intervention increases or decreases comfort for patients.*	2	5	Anticipated
		4	7	Concurrent

Code	Definition	Number of interviews	Number of text units	Temporal perspective
Prevention of transmission	The prevention of transfer of an infection from person to person. *Include statements referring to the influence of POC PCR-testing on transmission of respiratory infections within the healthcare setting.*	13	26	Anticipated
		26	60	Concurrent
Prevention of transmission population level	The prevention of transfer of an infection from person to person which has an impact on population wide occurrence of infection and resulting burdens. *Include all statements about how POC PCR-testing influences transmission and spread of COVID, Influenza or RSV on a population level.*	1	1	Anticipated
		5	6	Concurrent
Quality of care	The degree to which health services for individuals and populations increase the likelihood of desired health outcomes. *Include statements on how the quality of patient care is perceived to be altered by the use of POC PCR-testing.*	3	5	Anticipated
		2	3	Concurrent
Relative advantage	The degree to which a new intervention is superior to an existing one. *Include all statements that refer to how POC PCR-testing is perceived compared to other testing strategies*.	13	30	Anticipated
		26	72	Concurrent
Targeted patient management	The directed and efficient management of primary health care and access to other necessary medical or allied services for patients. *Include all statements that refer to how POC PCR-testing influences further patient management in form of admission, isolation or transfers as well as hygiene measures.*	13	21	Anticipated
		26	109	Concurrent
Time-to-Result	The interval from the first decision to perform a test to the diagnosis or secured absence of a certain disease in a patient. *Include statements about time-to-result and time-to-diagnosis and perceptions as to how this influences working procedures.*	10	13	Anticipated
		23	69	Concurrent

Code	Definition	Number of interviews	Number of text units	Temporal perspective
Treatment initiation	The first delivery of measures intended to improve the health outcome of a patient. *Include statements concerning the impact of the POC PCR-testing strategy on treatment initiation. Treatment being either a treatment for the respiratory infection or other treatment that might be affected by the COVID/Influenza status.*	5	18	Anticipated
		14	26	Concurrent
No unnecessary lack of treatment	The avoidable absence or postponement of treatment because of unclear infection status*. Include statements referring to the unnecessary lack of treatments such as chemotherapy due to unclear COVID-19 or influenza status.*	3	4	Anticipated
		2	2	Concurrent
**Self-Efficacy**	The participant's confidence that they can perform the behaviours required to participate in POC PCR-testing and achieve implementation goals.			
Indication	A medically valid reason to use a certain diagnostic tool. *Include statements referring to the perceived ability to decide on the indication of POC PCR-testing.*	2	3	Anticipated
		15	52	Concurrent
Perceived manageability	The perception of an intervention as possible to deal with or being controlled. *Include statements referring to the perceived manageability of performing the swabs and usage of the testing POC PCR-devices as well as carrying out the intended testing strategies.*	14	50	Anticipated
		26	142	Concurrent
Perceived manageability of problems	The capability of dealing with occurring problems related to the intervention. *Include statements on how problems concerning POC PCR-testing are perceived to be manageable or solvable.*	2	2	Anticipated
		9	18	Concurrent

Code	Definition	Number of interviews	Number of text units	Temporal perspective
Workflow	The sequence of operations and procedures in the healthcare setting. *Include statements about how the participant manages to include POC PCR-testing related procedures into their daily clinical workflow or how they feel the intervention influences their workflow.*	3	5	Anticipated
		13	17	Concurrent

Feasibility of POC PCR-testing

Code	Definition	Number of Interviews	Number of references
**Characteristics of Individuals**	The characteristics, assets and attitudes of the individuals in the healthcare setting of interest for POC PCR-testing.		
Knowledge & Beliefs about the Innovation	Individuals' attitudes toward and value placed on the innovation, as well as familiarity with facts, truths, and principles related to the innovation.	37	177
Other Personal Attributes	A broad construct to include other personal traits such as tolerance of ambiguity, intellectual ability, motivation, values, competence, capacity, and learning style.	13	23
Self-efficacy	The participant's confidence that they can perform the behaviours required to participate in POC PCR-testing and achieve implementation goals.	31	92
**Inner Setting**	The setting in which POC PCR-testing is implemented (our unit of analysis). In some settings there are multiple levels within the inner setting, namely the unit's healthcare team with local decisionmakers and the hospital with a taskforce or umbrella company in which the unit operates.		
Culture	Norms, values, and basic assumptions of a given organization (based on the Competing Values Framework). *Include statements related to the definitions of team culture, hierarchical culture, entrepreneurial culture and rational culture.*	15	63
Implementation Climate	The absorptive capacity for change, shared receptivity of involved individuals to an innovation, and the extent to which use of that innovation will be rewarded, supported, and expected within their organization. *Include statements regarding the general level of receptivity to implementing POC PCR-testing.*		
Compatibility	The degree of tangible fit between meaning and values attached to the innovation by involved individuals, how those align with individuals' own norms, values, and	31	136

Code	Definition	Number of Interviews	Number of references
	perceived risks and needs, and how the innovation fits with existing workflows and systems. *Include statements that POC PCR-testing did or did not need to be adapted as evidence of compatibility or lack of compatibility.*		
Opposition	Attitudes that go against or disagree with the intervention. *Include statements mentioning resistance from stakeholders to POC PCR-testing due to other priorities or competing interests.*	35	119
Relative Priority	Individuals' shared perception of the importance of the implementation within the organization. *Include statements that reflect the relative priority of POC PCR-testing, e.g., statements related to change fatigue in the organization.*	35	204
Tension for Change	The degree to which stakeholders perceive the current situation as intolerable or needing change. *Include statements that (do not) demonstrate a strong need for POC PCR-testing and/or that the current situation is untenable.*	26	103
Networks & Communications	The nature and quality of webs of social networks, and the nature and quality of formal and informal communications within an organization. *Include statements about general networking, communication, and relationships in the organization, such as descriptions of meetings and methods of keeping people connected and informed, and statements related to team formation, quality, and functioning.*	13	28
Readiness for Implementation	Tangible and immediate indicators of organizational commitment to its decision to implement an innovation. *Include statements regarding the general level of readiness for implementation.*	0	0

Code	Definition	Number of Interviews	Number of references
Access to Knowledge & Information	Ease of access to digestible information and knowledge about the innovation and how to incorporate it into work tasks. *Include statements related to implementation leaders' and users' access to knowledge and information regarding use of POC PCR-testing.*	18	31
Available Resources for Implementation	The level of resources organizational dedicated for implementation and on-going operations including physical space and time. *Include statements related to the presence or absence of resources specific to implementation of POC PCR-testing.*	3	3
Funding	Money provided by an organization or government for implementation and use of an innovation. *Include statements about (not) having adequate funding for POC PCR-testing to be implemented, embedded and sustained*.	3	3
General resources	Any resources needed to manage and maintain POC PCR-testing. *Include statements referring to the need of general resources for successful use of POC PCR-testing.*	9	14
Infrastructure	The resources required to support the initiative to be delivered such as buildings, office space, materials and supplies. *Include statements related to (lack of) available infrastructure needed for POC PCR-testing.*	30	89
Staff	Having a sufficient number of staff to meet the requirements of the initiative. *Include statements on (in-)sufficient staff resources for implementing and carrying out POC PCR-testing.*	15	35
Time	Stakeholders' availability of time for the intervention. *Include statements referring to (not) available energy and time to dedicate to POC PCR-testing.*	11	18

Code	Definition	Number of Interviews	Number of references
Support available	The presence of professional support if needed. *Include statements on support in the form of reminders, staff, technical and educations that is available to enhance delivery and maintenance of POC PCR-testing*.	6	9
Integration with existing programs and policies	The need to ensure an initiative was embedded into organisational structures, programmes and policies. *Include statements on how well POC PCR-testing can be integrated into existing programs, workflows and policies.*	10	22
Leadership Engagement	Commitment, involvement, and accountability of leaders and managers with the implementation of the innovation. *Include statements regarding the level of engagement of organizational leadership in POC PCR-testing.*	12	22
Structural Characteristics	The social architecture, age, maturity, and size of the organization. *Include statements about the structural characteristics of the organization of the ambulatory unit where POC PCR-testing is implemented.*	6	8
**Intervention Characteristics**	The key attributes of an intervention that influence implementation success.		
Complexity	Perceived difficulty of the innovation, reflected by duration, scope, radicalness, disruptiveness, centrality, and intricacy and number of steps required to implement. *Include statements regarding the complexity of POC PCR-testing itself.*	26	95
Cost	Costs of the innovation and costs associated with implementing the innovation including investment, supply, and opportunity costs. *Include statements related to the financial cost of POC PCR-testing and its implementation.*	20	54

Code	Definition	Number of Interviews	Number of references
Cost of Implementation	The financial cost of implementing an innovation. *Include statements related to costs of purchasing the testing device itself and logistic requirements needed to be satisfied in order to implement POC PCR-testing.*	4	8
Opportunity Cost	The extent to which benefits, profits, values have to be given up to engage in the intervention. *Include statements about loss of other benefits due to POC PCR-testing.*	14	25
Remuneration	The payment of the expenses incurred as a loss covered by an insurance policy. *Include mentions of receiving remuneration for performing the intervention from health insurance providers, and reimbursement of cost of implementing POC PCR-testing.*	6	15
Running Costs	The costs of maintaining and carrying out an already implemented innovation. *Include statements related to e.g. the cost per test, payment of new employees who perform the test, maintenance.*	12	15
Evidence Strength & Quality	Stakeholders' perceptions of the quality and validity of evidence supporting the belief that the innovation will have desired outcomes. *Include statements regarding awareness of evidence and the strength and quality of evidence, as well as the absence of evidence or a desire for different types of evidence, e.g. pilot results instead of evidence from the literature.*	9	19
Intervention adaptation and receptivity	The ability of an initiative to respond to change and adapt to fit with local contexts and requirements. *Include statements about adaptation of POC PCR-testing in the respective settings.*	4	9

Code	Definition	Number of Interviews	Number of references
Relative Advantage	The degree to which a new intervention is superior to an existing one. *Include stakeholders' perception of the advantage of implementing POC PCR-testing versus an alternative solutions and statements that demonstrate that POC PCR-testing is better (or worse) than existing programs.*	39	297
**Outer Setting**			
Awareness and raising the profile	Ensuring that stakeholders such as the community are aware of the initiative and its benefits and strategic steps are taken to raise the profile of the project to garner further support though media, marketing and publications. *Include statements about the measures taken and noticed by stakeholders to raise awareness of POC PCR-testing.*	0	0
External Policy & Incentives	External strategies to spread innovations including policy and regulations (governmental or other central entity of the healthcare system), external mandates, recommendations and guidelines, pay-for-performance, collaboratives, and public or benchmark reporting. *Include descriptions of external motivators and incentives influencing POC PCR-testing implementation as well as experienced, desired or feared impact of socioeconomic and political impact on POC PCR-testing practices.*	17	39
Needs & Resources of Patients	The extent to which the needs of patients, as well as barriers and facilitators to meet those needs, are accurately known and prioritized by the organization. *Include statements demonstrating (lack of) awareness of the needs and resources of patients. In addition, include statements that capture whether or not awareness of the needs and resources of those served by the organization influenced the implementation or adaptation of POC PCR-testing.*	18	42

Code	Definition	Number of Interviews	Number of references
**Process**	The process of connecting an intervention and setting with effective implementation.		
Engaging	Attracting and involving appropriate individuals in the implementation and use of the innovation. *Include statements related to engagement strategies and outcomes, i.e., if and how staff and innovation participants became engaged with POC PCR-testing and what their role is in implementation.*		
Formally Appointed Internal Implementation Leaders	Individuals from within the organization who have been formally appointed with responsibility for implementing an innovation as coordinator, project manager, team leader, or other similar role. *Include statements related to engagement strategies and outcomes, e.g., how the formally appointed internal implementation leader became engaged with POC PCR-testing and what their role is in implementation.*	15	27
Innovation Participants	Individuals served by the organization that participate in the innovation, e.g., patients in a prevention program in a hospital. *Include statements related to engagement strategies and outcomes, e.g., how innovation participants became engaged with POC PCR-testing.*	19	28
Key Stakeholders	Individuals from within the organization that are directly impacted by the innovation, e.g., staff responsible for making referrals to a new program or using a new work process. *Include statements related to engagement strategies and outcomes, e.g., how key stakeholders became engaged with POC PCR-testing and what their role is in implementation.*	13	34
Opinion Leaders	Individuals in an organization that have formal or informal influence on the attitudes and beliefs of their colleagues with respect to implementing the innovation. *Include statements related to engagement strategies and outcomes,*	1	1

Code	Definition	Number of Interviews	Number of references
	e.g., how the opinion leader became engaged with POC PCR-testing and what their role is in implementation.		
Quality of Staff	The capabilities, motivation, and skills of the engaged staff. *Include statements on which qualities and skills enable the engaged staff to carry out POC PCR-testing and statements about which qualities are needed.*	17	38
Reflecting & Evaluating	Quantitative and qualitative feedback about the progress and quality of implementation accompanied with regular personal and team debriefing about progress and experience. *Include statements that refer to the implementation team's (lack of) assessment of the progress toward and impact of POC PCR-testing implementation, as well as the interpretation of outcomes related to implementation.*	8	16
**Sustainability**	The ability to maintain programming and its benefits over time, the extent to which a newly implemented intervention is maintained or institutionalised within a service setting's ongoing stable operations	15	78
Initiative design and delivery	The way an Intervention is designed and delivered to stakeholders and intervention participants. *Include statements on how POC PCR-testing implementation was designed and delivered as well as suggestions for intervention design and delivery.*	12	49
Demonstrating effectiveness	The demonstration of effectiveness of POC PCR-testing strategies within the own organization. *Include statements about effects POC PCR-testing actually had in the institution while using the intervention.*	4	8

Code	Definition	Number of Interviews	Number of references
Program Drift	The phenomenon whereby deviation from manualized protocols in real-world delivery of interventions is expected to yield decreasing benefit for patients. *Include statements about e.g. deviations from POC PCR-testing SOPs or other unintended changes in testing strategies.*	9	18
Voltage Drop	The phenomenon in which interventions are expected to yield lower benefits as they move from efficacy to effectiveness and into real world use. *Include statements about observed decline in benefit of POC PCR-testing compared to expected benefit in the literature.*	0	0
Improvement methods	The use of improvement methods to support initiative success and sustainability. *Include statements about used and desired improvement methods to enhance POC PCR-testing benefits.*	10	27
Monitoring progress over time	The ability to monitor the initiative using standardised systems or mechanisms over time. *Include statements about measures taken or needed to monitor POC PCR-testing processes and quality.*	11	23
Outcome assessments	Real life practices to assess intervention outcome. *Include statements on internal assessment of achievement of intended intervention outcomes.*	2	2
The Problem	The recognition, concern and acceptance of a problem that supports an initiative to address it. *Include statements about persistent presence of awareness of the problems targeted by POC PCR-testing strategies.*	32	94
Training and capacity building	Orienting and training staff to be able to deliver the initiative successfully, as well as putting ongoing educational and skill building support in place for new workers.	17	71

Code	Definition	Number of Interviews	Number of references
	Include statements of current and desired training or engaging practices for POC PCR-testing.		
Negotiating initiative processes	The process in which the ground rules and necessities as well as responsibilities are defined by the organization. *Include statements about the (lack of) definition of rules and responsibilities before and during implementation of POC PCR-testing.*	6	13
Accountability of roles	Roles and responsibilities involved in the initiative are clearly defined and outlines with necessary distribution across teams as necessary so there is no reliance on specific individuals. *Include statements about (missing) clear accountability of roles in the process of POC PCR-testing implementation and maintenance.*	23	71
Belief in the initiative	The belief that, the initiative will be of value, it will produce the benefits intended and deliver the stated improvements to care. *Include statements about the belief of stakeholders that POC PCR-testing will produce the intended benefits.*	35	112
Defining aims and shared visions	Taking the time to define and understand what people want to achieve and why. *Include statements about POC PCR-testing aims that were defined before implementation and mentions of working with stakeholders to establish a shared aim and vision.*	6	8
Stable resources over time	The availability of resources that do not vary a lot and that will be available for long term use. *Include statements about stability of financial, time and staff resources over the course of POC PCR-testing.*	0	0

Code	Definition	Number of Interviews	Number of references
Stakeholder participation	Participation of those involved in the intervention after initial engaging process is terminated. *Include statements about stakeholder participation and participation strategies in POC PCR-testing in long-term execution.*	9	16
