# Supplementary material for: Implementation of Point-of-Care PCR-testing for the diagnosis of respiratory infections in vulnerable patient populations
Source: PLoS One. 2025 Jul 29;20(7):e0307621. doi: 10.1371/journal.pone.0307621 (PMC12306790; doi:10.1371/journal.pone.0307621)

Coding trees acceptability and feasibility

1. Coding tree for acceptability based on the Theoretical Framework of Acceptability (Sekhon et al., 2017).

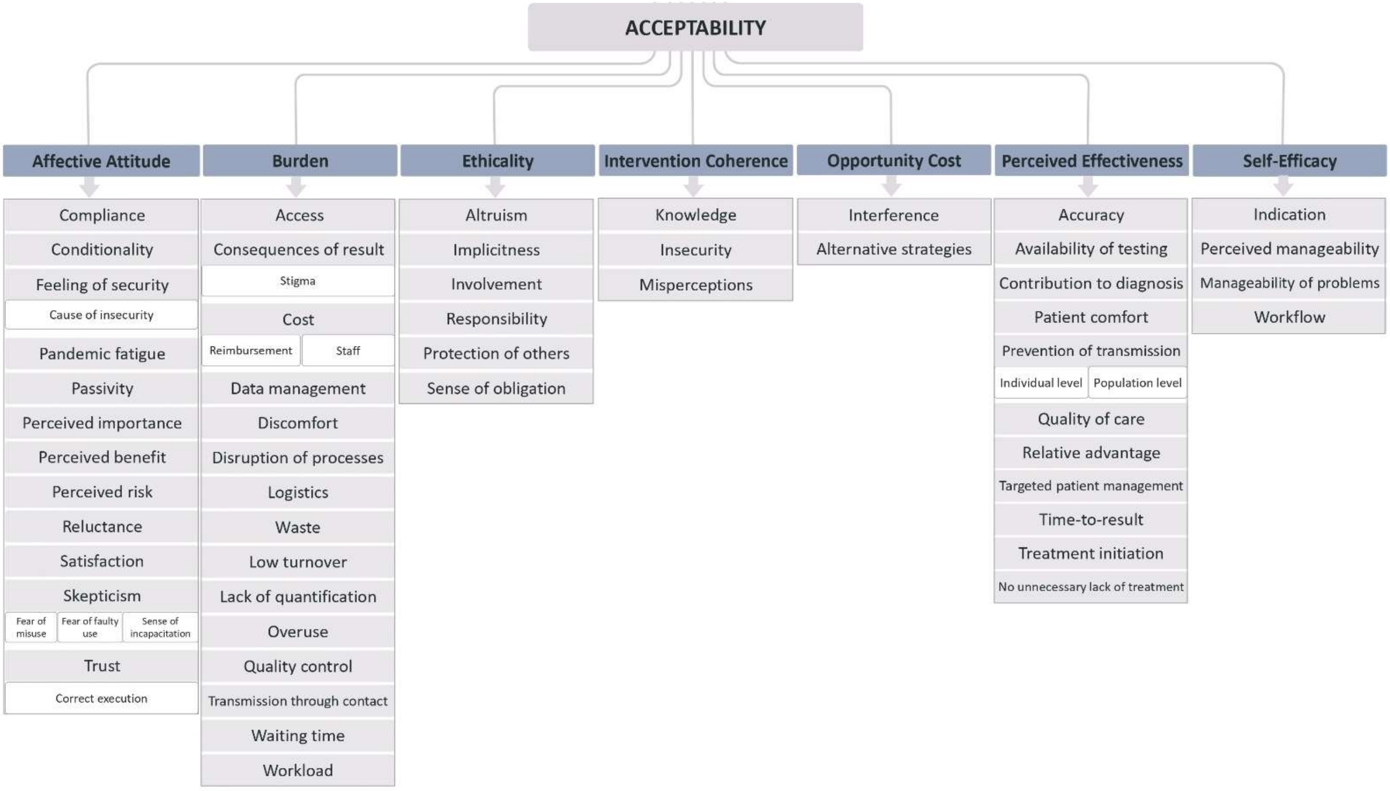

2. Coding tree for feasibility based on the Consolidated Framework of Implementation Research (CFIR, 2009) and the Consolidated Framework for Sustainability constructs in healthcare (Lennox et al., 2018).

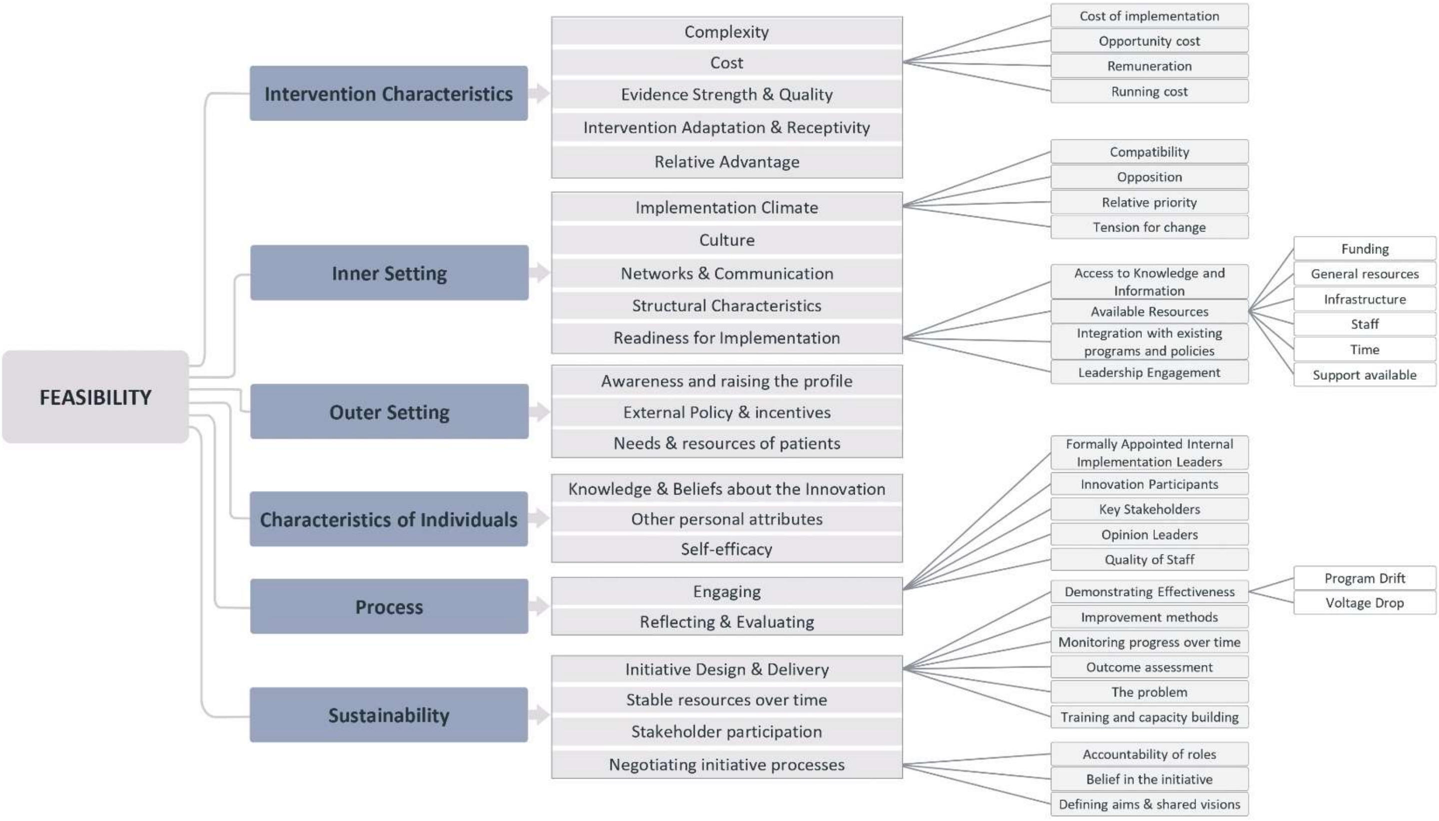

Supplement: S6 Fig — (PDF) [file pone.0307621.s006.pdf]
