# Supplementary material for: Implementation of Point-of-Care PCR-testing for the diagnosis of respiratory infections in vulnerable patient populations
Source: PLoS One. 2025 Jul 29;20(7):e0307621. doi: 10.1371/journal.pone.0307621 (PMC12306790; doi:10.1371/journal.pone.0307621)
Supplement: S7 File — (PDF) [file pone.0307621.s007.pdf]

Reflexivity

Reflexivity is about acknowledging the researcher's role in the research. As qualitative researchers, we are part of the research process, and our prior experiences, assumptions and beliefs influence the research process. In this statement we reflect on the ways in which we as researchers and the research process may shape the data collected, including the role of prior assumptions and experience.

Prior assumptions and experience

Within the context of the current study, there was direct contact with study participants, which evokes the need to consider the ways in which the researcher's interactions with participants might be influenced by their own professional background, experiences and prior assumptions. The interviewer (HT) was a last-year female medical doctoral student with extensive prior training in interviewing in a qualitative research context. There was no affiliation with the wards or ambulatory units of interest. Participants were either approached directly for the interview in their respective medical ambulatory setting, or a time and date for the interview was arranged beforehand via telephone call or e-mail contact. We conducted one interview per participant. Since participants were informed about the interviewer's professional role, an important question to consider before drawing conclusions from the data concerned whether or not knowing about the professional background could have impacted on participants' willingness to talk openly about experiences, or how this knowledge might have shaped what was said, such as an unconscious feeling that they should talk favourably about medical interventions. The interviewer addressed this in the beginnings of interviews.

After each interview a cover sheet with field notes, reflections on the specific interview setting, predominant themes and impressions on 'how the interview went' as well as considerations on emotional and other factors influencing the interview procedure or addressed themes was created for consideration in later analysis processes. Participants were not involved in study procedures after termination of the interviews.

Awareness of social setting and the social 'distance' between the researcher and the researched

The majority of interviews were conducted in the ambulatory units of interest in a booth or after hospitalization in a hospital room (for patients), or in the participants' respective bureaus or empty break rooms, depending on the current availabilities (for healthcare personnel and decisionmakers). One interview was conducted in a decisionmaker's home. Especially the breakroom setting produced a potential feeling of impeached confidentiality, which is why this setting was only chosen if no other options were available and with explicit approval of participants. In case of entering colleagues, the interview was interrupted until privacy was reinstalled. Interviews were all conducted face to face. Although the interview situations were entered as researchers, we were also mindful that we were potentially seen as part of the institution to which the testing unit was affiliated, which we tried to clear up before starting the interview, since this assumption might lead to participants feeling less able to talk freely e.g. about issues concerning their supervisors. Since especially patients are in a vulnerable situation at the time of visiting a healthcare facility, we explicitly explained their option to opt out of the interview in case they feel that they don't want to continue. In some settings (e.g. oncological patients) healthcare staff was asked beforehand who might be in a too vulnerable situation and headspace to approach.

Potential for psychological harm

The researchers involved in planning and conducting the interviews (HT, IM) were aware that focusing on the research topic could potentially provoke anxiety about COVID-19, influenza, or RSV infection in the respective health setting in the research participants (especially patients). Discussion guides were

pilot tested with an emphasis on non-harmful language and interview flow. At the end of each interview, the interviewer took time to ensure that participants were not feeling distressed by their participation. Throughout the interviews, none of the participants expressed newly evoked concerns or appeared to be distressed.

Fair dealing

One way of reducing bias in qualitative research is to ensure that the research design explicitly incorporates a wide range of different perspectives, so that the viewpoint of one group is never presented as if representing the sole truth about any situation, an analytic technique that was referred to as 'fair dealing' (Mays & Pope, 2000).

Our study was designed to elicit contributions from a broad range of stakeholders involved or potentially involved in POC PCR-testing. By including settings who had not yet adopted POC PCR-testing we aimed to include more sceptical point of views or to better illuminate reasons for non-adoption. During the analytic process no particular group's views were 'privileged' over those of others; that is to say, data analysis included a process of constant comparison between different accounts, to uncover similarities and differences, which were subsequently highlighted in the categorized data.

In the analysis, we sought to identify the views and experiences of individuals, as well as the majority, where these were divulged.

Awareness of wider social and political context

We discussed the fact that some decisionmakers were recruited from a hospital policy level (task force), or a professional laboratory organisation and might therefore show a strong commitment to a particular personal or political agenda or wish to raise issues that are only tangentially related to the research topic. We dealt with this by emphasizing the purpose of the research prior to the interview and through the questions and probes used. This strategy appeared to be successful in keeping participants engaged in the research process.

Collaboration in knowledge production

Collaborative research is highly valued for its ability to bring together multiple researchers with distinctive and specialist perspectives to tackle large or complex research problems.

Within the research team, we made an effort to work collaboratively in the collection, analysis, interpretation and reporting of the qualitative data, though individual involvement with the various stages of the research process necessarily varied. The two team members most closely involved in fieldwork (HT, IM) met regularly to discuss the progress of fieldwork and reflect on data collection; Meetings were held to identify the relevant emerging themes and codes and to discuss the construction of the codebook. At this crucial stage, input was sought from other members of the research team with more extensive experience of qualitative research (MC, JW) and with previous experience using the Framework approach (JW) to assist with upcoming considerations concerning further participant recruitment and for discussing codes and solidifying the coding framework. This endeavour resulted in an analytic strategy that was informed by insights from team members with a broad understanding of the research field and methodological issues, and those with field-based contextual and experiential understanding. Coding was carried out by the same researcher who had conducted the interviews (HT) in two rounds to increase coding reliability.
