# Supplementary material for: Implementation of Point-of-Care PCR-testing for the diagnosis of respiratory infections in vulnerable patient populations
Source: PLoS One. 2025 Jul 29;20(7):e0307621. doi: 10.1371/journal.pone.0307621 (PMC12306790; doi:10.1371/journal.pone.0307621)
Supplement: S8 Table — (PDF) [file pone.0307621.s008.pdf]

Construct	Perceptions of stakeholders[a]	Construct	Perceptions of stakeholders
**Burden & Opportunity cost**<br>*the amount of effort required and the extent to which benefits, profits, values have to be given up to engage in the intervention*	***Cost***<br>*Main focus of decision makers*<br>- Financial: cost of device, cartridges and technical integration (+)[b], difficulty of reimbursement (+), costly overuse of available device<br>- Personal and technical resources<br>- Need of a profit for health institutions<br>***Workload*** (+)<br>*For healthcare personal*<br>- Time per test and documentation, training of staff, quality control and laborious hygiene measures of isolation/cohorting (+)<br>- Increased workload through double testing (POC and rt- PCR)<br>- Waiting time, one POC testing device with low turnover as “bottle neck” vs time sensitivity of testing<br>- Implementation effort for protection of few<br>- Better effort-benefit ratio in panel tests<br>***Discomfort***<br>*of nasopharyngeal swab (+) and isolation*<br>***Logistics***<br>- Separation of patient streams, testing room (+)<br>*Transmission through testing on site, contaminated testing site*<br>-Ordering and storage of testing supplies, waste disposal, data management and result availability<br>*Ensuring availability and accessibility of device*<br>***Interference***<br>*of testing with other patient care “within reasonable bounds”*<br>***Consequences of positive result*** *“don’t want to know”*<br>- Need to isolate (+), loss of other treatment (e.g. chemo), postponed diagnostics<br>- Finding a hospital bed for positive patients (+)<br>***Culture***<br>*Need for approval by decisionmakers and laboratories*<br>- Central laboratories trying to retain specialized diagnostics	**Affective attitude**<br>*how an individual feels about the intervention*	***Perceived Importance***<br>*Dependent on perception of vulnerability and risk (+)*<br>- POC testing as only viable option in vulnerable populations<br>*Necessity for further patient management and treatment (+), “lifesaving”*<br>- Wish for broader and generalized implementation<br>- Sufficiency of current strategy (+)<br>*such as Ag-tests + PCR, CRP testing, vaccination, external testing or basic hygiene measures*<br>*Especially in participants with low intervention coherence and for influenza*<br>- Conditionality (+)<br>*Support depending on pandemic situation, indication, setting, compatibility, effort, cost and relative advantage and quality of staff.*<br>***Satisfaction and Trust***<br>*POC testing as “little treasure”, ”blessing” or “appreciated gift”*<br>- Generally high level of approval and confidence in testing procedure, device, correct handling, result accuracy (+) and intervention benefit<br>*Aligning oneself with the opinions of experts*<br>- Fear of incorrect execution of testing procedures and errors during data management<br>*False sense of security*<br>***Feeling of security***<br>*through fast knowledge of diagnosis and reliable results vs no absolute safety and sense of insecurity through too frequent testing*<br>- Having “something tangible” to protect yourself and others (+), regaining control<br>***Reluctance*** (+)<br>*before and in beginning of the implementation process; mostly in screening scenario.*<br>- Taking on the higher workload (+), fear of becoming a test centre<br>- Scepticism about accuracy, manageability, benefit and evidence<br>- Pandemic fatigue and fatalism<br>- Feeling monitored and incapacitated, fear of stigma of positive result
**Ethicality**<br>*the extent to which the intervention has good fit with an individual’s value system*	***Medical appropriateness and implicitness*** *„the means of choice”*<br>- “Indispensable” especially in vulnerable groups and symptomatic patients (+)<br>*Quality assurance and integrability must be guaranteed.*<br>***Sense of responsibility***<br>*towards the general public and other patients*<br>- Evaluation as obligatory vs unwillingness to be tested<br>- Perception as „additional burden“ vs „additional task”	**Intervention coherence**<br>*the extent to which a participant understands the intervention*	***Comprehension***<br>*of risk, infection, test characteristics and usage, quality assurance, standard operating procedures (+)*<br>- Perceived as essential in users<br>*Increased through official initial training*<br>- Higher in diagnostics experts and nurses (procedural), lower in patients and doctors<br>- Generally low level of interest, known evidence (+) and reflection

	- Necessary investment for more safety<br>***Altruism***<br>**-** Protection of others (+), an inconvenience to be endured for patient well-being and the greater good<br>***Compliance (+)***<br>*Estimated low in others and children*<br>- Passive compliance with testing strategy and external regulations vs active contribution and wish for greater involvement in intervention design	*and how it works*	- Mostly unofficial sources of information<br>***Misperceptions***<br>*about test characteristics: necessity of a laboratory, confusion with Ag-testing, accuracy, treatment options and availability*<br>- Contradictions regarding current strategy (+) and decision making<br>***Insecurities***<br>*about accuracy, cost (+), evidence, consequences of testing, potential sources of errors and evaluation of the intervention quality and effectiveness*<br>- due to lack of information
**Self-efficacy**<br>*the participant's confidence that they can perform the behaviours required to participate in the interventions*	***Perceived Manageability***<br>- Ease of use (+), reliability and simplicity of intervention: "self-explanatory" and "fool proof"<br>*Confidence in proper handling of devices (+) and methodological expertise*<br>- Requirements<br>*Adequate training (+), hygiene awareness, professionalism and accountability of roles, documentation, administration, and standard operating procedures.*<br>- Perceived struggles<br>*Lack of staff (+), patient compliance, compliance with hygiene measures, deficient swab techniques, invalid results, forgetting running tests, prevention of abuse by other departments and consequences of positive results*<br>- Quick resolution of past problems<br>*Through learning by doing, appointed specialists and device support*<br>- Testing as insignificant inconvenience in situation of illness<br>***Workflow***(+)<br>- Good fit of testing procedures with usual workflow, "sure-fire success" vs disruption and delay of already tense processes<br>- sufficient flexibility and time resources of patients<br>- symptomatic and contact testing as most viable strategy; high frequent testing e.g. in screening not manageable<br>***Indication***<br>*Wish for more information about reasoning behind indication.*<br>- Confidence in correct indication of testing (+) or knowledge of SOPs vs conflicts about correct indication<br>*"no self-service store", requirement of good communication*	**Perceived effectiveness**<br>*the extent to which the intervention is perceived as likely to achieve its purpose*	***Testing Characteristics***<br>-High accuracy (+)<br>*compared to antigen tests, control via rt-PCR not perceived as necessary, mostly anecdotal evidence, reservation about isothermal panel tests*<br>- No quantification<br>*Need of Ct value for end of isolation of positive patients*<br>- Short time-to-diagnosis (+)<br>*compared to rt-PCR in the laboratory, fast confirmation of positive antigen tests*<br>-Availability of testing and results, no sample transport<br>-Benefit of performing a respiratory panel test (COVID, Influenza, RSV)<br>*Time saving and clear diagnosis.*<br>- Automated device with relatively low binding of personnel<br>***Consequences of testing***<br>- Targeted patient management (+)<br>*Improved quality of care: cohorting, facilitation and acceleration of following patient transfers and emergency interventions, convenience of on-site testing for patients, relief for healthcare workers.*<br>- Contribution to diagnostic process<br>*Enabling decision making (e.g., about admission), incidental findings or exclusion of infection, less burden due to false positive cases, faster treatment initiation, stop of immunosuppressive treatment during infection*<br>- Prevention of transmission (+) and intrahospital outbreaks<br>*Between patients and to healthcare workers through adequate hygiene measures, mostly speculative*<br>- "Would not make a difference"<br>*No impact on treatment for many, risk of outbreak already well controlled, no population level benefit*

[a] stakeholders are decisionmakers, healthcare workers and patients in a paediatric emergency care unit, who are involved with POC PCR testing for respiratory infections
[b] most prevalent statements are marked with (+)
