# Supplementary material for: Implementation of Point-of-Care PCR-testing for the diagnosis of respiratory infections in vulnerable patient populations
Source: PLoS One. 2025 Jul 29;20(7):e0307621. doi: 10.1371/journal.pone.0307621 (PMC12306790; doi:10.1371/journal.pone.0307621)
Supplement: S9 Table — (PDF) [file pone.0307621.s009.pdf]

**Intervention Characteristics**	**COMPLEXITY**<br>- Ease of use, reliability and simplicity of POC PCR-testing<br>*"self-explanatory" and "fool proof", very rare occurrence of intervention related problems*<br>- low binding of personnel<br>*ca 5 min active work, mostly automated*<br>- perceived unnecessary disruptiveness<br>*double testing, multiple documentation and lengthy validation process when starting a new lot*<br>**RELATIVE ADVANTAGE**<br>***Existence***<br>-High accuracy<br>*compared to antigen (Ag)-tests, control via laboratory PCR not perceived as necessary, mostly anecdotal evidence (COVID), reservation about isothermal panel tests*<br>- Short time-to-result<br>*compared to rt-PCR in the laboratory, fast confirmation of positive Ag-tests*<br>-Availability of testing and results, no sample transport<br>-Additional benefit of performing a respiratory panel test (COVID, Influenza, RSV)<br>*Time saving and clear diagnosis*<br>***Perceived advantage***<br>- Targeted patient management<br>*Improved quality of care: cohorting, facilitation and acceleration of following patient transfers and emergency interventions, relief for healthcare workers.*<br>- Contribution to diagnostic process<br>*Enabling decision making (e.g. about admission), incidental findings or exclusion, less burden due to false positive cases, treatment initiation, stop of immunosuppressive treatment during infection*<br>- Prevention of transmission and intrahospital outbreaks<br>*Between patients and to healthcare workers through adequate hygiene measures, mostly speculative*<br>***Lack***<br>- No quantification<br>*Decreased usefulness for entisolation, positive results in patients with high Ct-values.*<br>- No impact on treatment for many, risk of outbreak well controlled, no population level benefit.<br>**COST**<br>*Main focus of decision makers, need for profitableness of health institutions.*<br>***Implementation***<br>- Cost of device and technical integration<br>*Need for regular testing to make the purchase worthwile*<br>***Running Cost***<br>- Cartridges: 2x price of laboratory PCR, costly overuse of available device<br>- Personal and technical resources<br>*Additional costs through double testing (POC and rt-PCR)*<br>***Remuneration***<br>- Difficulty of reimbursement<br>*via health insurance providers (e.g. only accredited laboratories) or hospital administration, lack of financing*<br>***Opportunity Cost***<br>- Interference with other diagnostic and therapeutic measures "within reasonable bounds"<br>*medical emergencies remain first priority*<br>- High quality of laboratory testing<br>**EVIDENCE STRENGTH AND QUALITY**<br>- Accuracy tested by producers and in independent studies<br>*High perceived need for outcome related evidence: e.g. number-needed-to-treat, infection prevention and evidence for secondary cost saving for the health system*<br>**INTERVENTION ADAPTION AND RECIPTIVITY**<br>- Easy adaptation of POC PCR-testing workflow to different respiratory viruses<br>*Choice of tested respiratory infections (RSV, Influenza COVID) according to clinical need*<br>- Flexible strategy through changing of SOPs<br>*Indication and frequency of testing according to pandemic status and external demands*<br>- Intervention adapted as new standard for clinical decision making
**Inner Setting**	**CULTURE**<br>*According to the Competing Values Framework*

	- Mixed internal and external focus<br>- Centralization of decision-making (e.g. task force, shareholders or medical directors) with little user involvement, formalized and structured processes<br>*Hierarchical organization and clear accountability of roles vs flexibility of staff, cooperation and intervention adaptation*<br>**IMPLEMENTATION CLIMATE**<br>***Opposition***<br>*Mostly anticipated*<br>- Patients: discomfort of swab, general scepticism, pandemic fatigue, fear of incorrect execution or delayed treatment, low perceived necessity (panel tests as overdiagnistics), sense of insecurity and incapacitation through too frequent testing, consequences of positive result (delay of chemotherapy, isolation /quarantine)<br>- Healthcare workers: additional workload especially in frequent testing, lack of involvement in initiative design, sufficiency of ag-tests<br>*Mostly when having to tests for others*<br>- Decisionmakers: cost vs need for profitableness, disruption of processes, delayed patient transfer, no isolation capacity, transmission through contact, waste production, organisational burden, leaving diagnostic focus in the laboratory<br>***Compatibility***<br>*Values:* Medical appropriateness and implicitness*:* “Indispensable” especially in vulnerable groups, ERs and symptomatic patients<br>*No excessive, constant testing, quality assurance and integrability must be guaranteed.*<br>-Responsibility of healthcare institutions for safety and best practice in diagnostics and treatment of their patients<br>*Testing procedures:* “Fits well”, effort within „reasonable and easily manageable limits“<br>*Same procedures as other testing (eg. Rt-PCR, Ag-tests), familiarity with POC devices, being “used to it”, no difference in processes noticed by patients, perception as routine*<br>- vs time effort needs to be depicted in workflow<br>*especially in frequent testing, testing for others or tense staffing*<br>- Not compatible for screening e.g. in dialysis unit (cohorted schedules)<br>*Exchange with users perceived as necessary for good compatibility of testing strategy*<br>*Consequences:* difficulty handling positive patients vs “always had struggles with isolation”, “nothing new”<br>*Infrastructure:* Needed logistics are compatible with existing premises, compatibility with hospital information system<br>***Relative Priority***<br>*Dependent on perception of vulnerability and risk, perceived effectiveness as well as pandemic status*<br>- POC testing as only viable option in vulnerable populations upon suspicion<br>*Necessity for further patient management (e.g. admission to surgery or wards), diagnostics and treatment (e.g. indication for Paxlovid/ pausing immunosuppressive treatment), trying to keep vulnerable wards “clean” (e.g. haemato-oncologic ward or ICU)*<br>*Readiness to accept discomfort / expenses for more safety “you can’t have everything”*<br>- vs passive acceptance<br>*“would not bother me“, “no obligation”*<br>- Conditionality<br>*Screening not perceived as necessary. Panel test especially necessary for differentiation between COVID and influenza in winter.*<br>- Patient treatment always as priority<br>***Tension for Change***<br>*Strong fluctuations, dependent on perceived risk of infection or severe course of disease and pandemic status*<br>- Need for better infection control<br>*Fear of transmission, infection of staff and consecutive absence, exposure due to late rt-PCR results, lack of trust in Ag-test results, delayed increase of CRP*<br>- Insecurities in handling patients with unclear infection<br>*Needed isolation but no capacity for isolating all suspected cases, decisions on chemo continuation*<br>- Inconvenience of alternatives<br>*External testing and other hygiene measures such as ventilation in winter, prolonged opening hours due to lack of spatial capacity for physical distancing, incorrect position of the mask in elderly patients*<br>- Vs sufficiency of current strategy for control of infection

	such as Ag-tests + PCR, CRP testing, vaccination, external testing or basic hygiene measures, isolation and treatment based on Ag-testing, sufficiency of next-day results in dialysis scenario.<br>- COVID not seen as a persisting problem, fewer severe courses of disease<br>**READINESS FOR IMPLEMENTATION**<br>***Access to Knowledge & Information***<br>- Patients: Information upon request through staff<br>*Generally low level of information, wish for more transparency*<br>- Healthcare workers: Methodological expertise obtained in initial introduction by the producer or through colleagues, learning by doing, little access to scientific background knowledge through official sources<br>*Wish for information on clinical impact and more official training to avoid passed on sources of error vs sufficiency of snowball system*<br>- Decisionmakers: Knowledge obtained from colleagues, lecture of medical journals (Ärzteblatt) and consultation with laboratory staff<br>*Implementation as fast decision process with limited access to information*<br>***Available Resources for Implementation***<br>- Funding: through cost centre of university hospital, holding company or research projects<br>- General resources: limited availability of testing devices and resources (e.g. cartridges, reagents)<br>- Infrastructure: Need for a testing room with windows for ventilation, possibility to separate patient streams and isolate, dirty-work area for testing device and procedures with short distances between patient care and testing area, storage room for materials, disposal of infectious waste<br>*Mostly good fit of existing infrastructure with POC PCR requirements*<br>- Staff: Need for staff for testing, organisational aspects (e.g. ordering of material)<br>*general lack of healthcare staff, limited capacity for additional tasks, low device turnover leads to higher personnel binding than laboratory testing*<br>- Time: Need for 3-5min per test, 15 min waiting time for patients, 30 min waiting for automated quality control, secondary time saving through targeted management<br>*generally limited time capacity*<br>- Availability of support: easy contact with producer customer service or leasing laboratory Internal support through medical technicians and appointed experts<br>***Integration with existing programs and policies***<br>- Easy integration of testing procedures with usual workflow and current testing strategy<br>*same "handgrip", implementation simply seen as an expansion of testing strategy*<br>- Expensive automated integration of results into hospital or practice information system, manual population of software with the results possible<br>***Leadership engagement***<br>- High level of involvement of some key figures: Nursing directors and senior physicians, laboratory advisors of the taskforce<br>*Need for engagement of cost centre*<br>- Active participation of healthcare managers to shape the testing indication<br>- Low level of involvement of some clinical directors not involved in the implementation<br>**STRUCTURAL CHARACTERISTICS**<br>***Ambulatory units (university hospital)***<br>- Interdisciplinary task force as main decisionmaker, central cost centre, senior physicians and care managers as heads of department with a big team of healthcare workers.<br>***Medical care centres***<br>**-** Centres with several medical directors under a holding company with centralized management. Moderate sized team of healthcare workers.<br>**NETWORKS AND COMMUNICATION**<br>- Communication of implementation via e-mail, information sheet and verbal announcement in team reunion<br>***Network***<br>- Little contact to central laboratories, communication with manufacturer via appointed contact person, verbal exchange between staff and supervisors.<br>- Bigger suggestions for improvement via official requests<br>***Communication of results***<br>- Via phone calls or in person, documentation on paper or information system

**Outer Setting**	**AWARENESS AND RAISING THE PROFILE**<br>- Advertisement of POC PCR-testing by laboratory enterprises, introduction into ambulatory units as part of healthcare research<br>**EXTERNAL POLICY AND INCENTIVES**<br>***Compliance with governmental regulations***<br>- Quality control: compliance with the Rili-BÄK (e.g. obligatory participation in ring trials), compliance with infection protection and medical product law (e.g. reporting of positive COVID cases, training of staff)<br>- Institutional political decision-making concerning sites of implementation and confirmation of results via rt-PCR (leaving focus on central laboratory testing)<br>***Incentives***<br>- Reimbursement by health insurance providers, financing of testing devices in health care research projects<br>- Admission requirements of other health institutions (e.g. negative Sars-CoV-2 PCR)<br>***Demands***<br>- Wish for a consistent, general approach with non-compulsory guidelines including target groups and indication as well as financing<br>**NEEDS AND RESOURCES OF PATIENTS**<br>- Awareness of patients' need for protection, fast diagnosis and treatment<br>*willingness to take on workload and hygiene measures for the wellbeing of patients if necessary, testing as a means to reduce patients' discomfort due to other hygiene measures*<br>- Consideration of patients' time resources and of special needs of infants<br>- Sparing patients from the burden of false positive results<br>*e.g. rescheduling of diagnostic or therapeutic appointments such as chemo therapy*<br>- Need for on-site testing in sick patients<br>- Consideration of impact of testing effort on other patients needs
**Characteristics of the Individual**	**KNOWLEDGE & BELIEFS**<br>- Good knowledge of risk, infection, testing characteristics and handling and SOPs<br>*Perceived as essential in users and increased through official initial training*<br>- Higher in diagnostics experts and nurses (procedural), lower in patients and doctors<br>- Generally low level of interest, known evidence and reflection<br>- Misperceptions about test characteristics *(necessity of a laboratory, confusion with Ag-testing, accuracy, treatment options and availability)*<br>- Contradictions regarding current strategy and decision making<br>- Insecurities due to lack of information<br>*about accuracy, cost, evidence, consequences of testing, potential sources of errors and evaluation of the intervention quality and effectiveness*<br>**SELF EFFICACY** *(same as in acceptability)*<br>- High perceived manageability<br>*Confidence in proper handling of devices and methodological expertise, importance of adequate training, hygiene awareness, professionalism and accountability of roles, documentation, administration, and standard operating procedures*<br>- Perceived struggles<br>*Lack of staff, patient compliance, hygiene measures, deficient swab techniques, invalid results, forgetting running tests, prevention of abuse by other departments and consequences of positive results, "huge effort" in view of emergency care and in sum with all e.g. COVID measures*<br>- Quick resolution of past problems<br>*Through learning by doing, appointed specialists and device support*<br>- Patients: Sufficient time, testing as insignificant inconvenience in situation of illness<br>***Workflow***<br>- Good fit of testing procedures with usual workflow, "sure-fire success" vs disruption and delay of already tense processes<br>*Overall testing as "easy to compensate", through "clever timing of steps"*<br>- symptomatic and contact testing as most viable strategy; high frequent testing e.g. in screening not manageable<br>***Indication***<br>*Wish for more information about reasoning behind indication.*<br>- Confidence in correct indication of testing or knowledge of SOPs vs conflicts about correct indication

	'no self-service store', requirement of good communication<br>**OTHER PERSONAL ATTRIBUTES**<br>*See Ethicality in Acceptability*
**Process**	***Implementation***<br>- quick and unproblematic, "technically feasible", add on to previous practices<br>*Partly facilitated through use of other cartridges in already available device*<br>- classic implementation steps<br>*choice and purchase of device, material orderings, staff training, logistic setup, integration into information system and quality assurance practices (partly automated)*<br>**ENGAGING**<br>***Formally appointed implementation leaders***<br>- Officer for medical devices (healthcare worker or MFA/MTA) with official training by the producer, license to train others, knowledge of technical details and contact to the producer's customer service.<br>*Volunteered staff, MPG (German Medical Devices Act) delegate or staff with preceptor certificate*<br>- Delegation of upcoming tasks (e.g. ordering of materials, quality control) to suitable staff<br>- Laboratory delegate for choice and ordering of testing devices and coordination of implementation<br>***Innovation participants (patients)***<br>*No specific engaging strategy*<br>- Performance of tests, detailed explanation only upon demand<br>*Perception as "standard"*<br>- Patients are asked to participate in form of signalling symptoms before or upon arrival<br>***Key stakeholders***<br>- Engaging of healthcare and laboratory staff through official short announcement of new testing device and strategy<br>*after decision making, generally good reception.*<br>- Official training by the producer or via snowball system after initial training to implement correct procedures and documentation<br>***Opinion leaders***<br>- Involved laboratory staff encouraging implementation.<br>***Quality of staff***<br>- Well educated healthcare staff with the necessary skills to indicate and perform testing procedures are available, "used to it"<br>*Importance of medical background, correct swabbing technique und understanding of hygienical concepts, unqualified helpers as "no go"*<br>- Different habits of using the device leading to malfunction and therefore loss of time<br>*especially when external staff performs tests*<br>- Execution of swabs perceived as well done compared to external testing sites<br>**REFLECTING & EVALUATING**<br>- Intermediary for surveillance who reports testing quality to decisionmakers<br>- Care managers involved in discussions but not decision-making<br>*"proactively setting incentives" necessary*<br>- Low involvement and feedback of users and lack of influence in big hospital units vs better level of involvement in smaller units<br>- For giving feedback active contacting of superiors is needed, no fixed structures available
**Sustainability**	**STAKEHOLDER PARTICIPATION**<br>- Testing procedures perceived as normal and standard, approval of SOPs and guidelines, increase of initiative acceptance over time<br>- Part of regular processes with autonomous indication by healthcare workers or doctors<br>- Questioning of long-term indication in view of decreasing carefulness in public<br>**INITIATIVE DESIGN AND DELIVERY**<br>*Standardized implementation and coordination over several sites perceived as desirable*<br>- Quick design and implementation, "a few weeks"<br>*Surprise and limited involvement of future users, time sensitivity due to pandemic status*<br>- Consideration of needed specific workflows, local logistics and technical conditions<br>*Organisation of ordering structures and staff training mechanisms*<br>- Successive development of SOPs or internal conventions<br>*with clear indication (e.g. upon suspicion or external request)*

	- Suggestion of pilot project to assess necessary workflow structures and clinical outcome<br>***Demonstrating effectiveness***<br>*Broadly assumed effectiveness for prevention of transmission without actual evidence / quantification*<br>- No outbreaks<br>- Improved patient management<br>*Faster patient transfer and more efficient bed occupancy, facilitation of further steps (isolation)*<br>***Program Drift***<br>- testing planned in separate room but in reality, frequently in therapy room<br>- frequent performance of tests without explicit indication<br>*Control of adherence to testing conventions and SOPs to reduce workload and cost*<br>- Persisting use at one's own discretion "not getting out of hand"<br>*Increasing use due to availability of testing or lack of knowledge of current SOPs, no standardized control of correct indications, trust in staff*<br>***Improvement methods***<br>- Technical and logistical improvements<br>*Repairs, close logistic position of the testing device (to patient care site), tasks delegation to specific responsible*<br>- Testing strategy<br>*Creation or adjustment of SOPs according to current need and to be more practicable, establishing clear documentation processes and quality control measures, expansion of testing spectrum from single to panel tests ("only one swab")*<br>- Handling<br>*Search for error sources and follow-up training*<br>***Monitoring progress over time***<br>*Monitoring of effectiveness and correct handling of devices through implementation leaders*<br>- Quality Control<br>*Documentation of staff training and compliance with quality control standards*<br>- Outcome assessment<br>*Documentation of realized tests, partly assessment of accuracy compared to rt-PCR, no or pending systematic outcome assessment (eg. NNT)*<br>***The problem***<br>- Recognition of hematooncological, dialysis, infant and elderly patients and patients with immunosuppressive treatment as at risk, fear of catching COVID/influenza during therapy phase<br>*more safety necessary in health care centres frequented by this collective vs decreasing risk with new variants*<br>- Anecdotes about previous healthcare acquired infections due to insufficient standard testing strategy or severe cause of disease vs no memory of any setting related infections<br>- Long exposure in chemo or dialysis room or hospital rooms vs perceived lower risk compared to other public spaces<br>- Difficult clinical differentiation between COVID, influenza and less relevant respiratory infections, time lag of CRP elevation and low sensitivity of Ag-testing<br>- Need for fast diagnosis and treatment initiation among immunosuppressed patients, fast result required for admission to surgery, patient transfer and cohorting<br>- Vs lack of concern for infection in non-vulnerable populations, some see current strategy as sufficient or no risk for severe cause of disease<br>*through vaccination or because of previous experience*<br>- Concern about aggravated lack of staff due to transmission<br>Acceptance: COVID, influenza and RSV as healthcare challenges that need to be managed<br>***Training and capacity building***<br>- Training of stakeholders see "Process – Stakeholder Engagement"<br>- Follow-up training after occurred problems<br>- Assisting healthcare personnel from other units performing the tests for their patients<br>*experience of device malfunctioning after use by non-qualified staff*<br>- Aim of standardized implementation in several settings in case of scale up<br>- Necessity of specific personnel for high frequent testing<br>**NEGOTIATING INITIATIVE PROCESS**<br>- Main decisionmakers: medical directors, commercial management, hospital management<br>*Inclusion of experienced experts (laboratory) in initial decision process and of clinical staff in decisions on testing indication*

	- Discussion about necessary means and procedures to maintain hygiene standards, agreeing on reasonable sites of the testing device, initial SOPs, indication and eligible testers per device to prevent overuse<br>***Accountability of roles***<br>*Clear distribution of responsibilities (more necessary the bigger the institution)*<br>- Determination of testing strategy: *care managers, medical directors, shareholders, task force*<br>- Initial instruction and technical implementation: *medical technicians, producer, laboratory staff*<br>- Indication and decisions on consequences of result: *doctors, healthcare workers*<br>- Execution, documentation and communication of results: *healthcare workers, medical assistants*<br>- Logistics such as ordering of material and patient transfer: *senior physician, appointed healthcare workers, supply assistant*<br>- Device proficiency and customer service contact: *appointed officer for medical devices*<br>- Quality control and problem management: *care managers, appointed healthcare workers*<br>***Belief in the initiative***<br>*POC testing as "little treasure", "blessing" or "appreciated gift"*<br>- Generally high level of approval and perceived effectiveness<br>*often belief in effectiveness without clear reasoning, feeling of safety, limited belief in effectiveness of influenza POC PCR-testing (anticipated)*<br>- Trust in prevention of transmission (+) and intrahospital outbreaks, protection of vulnerable groups<br>*Simple intervention that achieves its goal, intervention perceived as "progress"*<br>- Vs "Would not make a difference"<br>*no complete safety but POC test as "a good start" or no effect on outbreaks perceived*<br>- Test as means to become capable of acting and relief<br>- Limited belief in impact on treatment vs evaluation as "life saving" through faster treatment initiation<br>***Defining aims and shared visions***<br>- Enabling timely testing of patients, protection of and adherence to SOPs<br>- POC PCR results as standard for further patient management<br>- Optimization of the entire healthcare structure and management by using POC PCR
