# Supplementary material for: Implementation of Point-of-Care PCR-testing for the diagnosis of respiratory infections in vulnerable patient populations
Source: PLoS One. 2025 Jul 29;20(7):e0307621. doi: 10.1371/journal.pone.0307621 (PMC12306790; doi:10.1371/journal.pone.0307621)
Supplement: S10 File — (PDF) [file pone.0307621.s010.pdf]

Citations in the original language

(in order of appearance in the manuscript)

[Also die aktuelle Vorschrift was jetzt die Patienten erstmal betrifft ist immer noch] Doppeltestung (*H*, E)

Standardschritte (*H*, D1)

Wir wussten, dass es kommt ungefähr vielleicht zwei, drei Wochen davor. Aber da war das ja schon beschlossene Sache. Insofern hätte man da jetzt nicht wirklich großartig was machen können. (*H*, E)

[Das ist eine] rein politische Entscheidung. (*L*1)

[Das steht bei uns auf Station, im Stationsstützpunkt, zugänglich für alle…] Kommt jeder zu jeder Zeit dran. (*H*, E)

[Wenn man das vorher testen kann, schützt das natürlich auch die anderen, die dann mit einem ja-... ich meine] hier im Zimmer ist man schon eng zusammen. (*P*, P)

[Da wir aber halt viele Patienten hatten, die respiratorische Infekte hatten und] wir jetzt nicht 42 Betten zu 20 machen können, weil jeder alleine liegt, [nutzen wir das immer in dem Moment, wo wir das Gefühl haben, dass jemand einen viralen Infekt hat, den wir darüber erkennen können.] (*D*, D1)

Der Patient musste nicht wieder nach Hause gehen, nen neuen Termin ausmachen, alles wieder neu organisieren. (*H*, P)

Sie haben das Ergebnis und Sie können entscheiden, Chemo ja oder nein. (*D*, O2)

[Andererseits,… klingt jetzt vielleicht doof, manchmal] wollen wir das gar nicht wissen. (*D*, P)

Wahrscheinlich mehr auch aus politischen Gründen, ja, also eine gewissen Grundversorgung sicherstellen, aber doch auch so den Fokus auf dem Labor belassen. (*L*1)

Hier sind ja sehr kranke Patienten, die ein super hohes Risiko haben. Und bei denen kann das Virus ja absolut auch tödlich verlaufen. (*P*, D1)

[Das ist absolut] nicht wegzudenken. (*D*, O2)

[Ja, das ist für den einen oder anderen dann sicher] lebensrettend (*D*, O1)

[Und wir haben das im letzten Jahr ja durchexerziert mit Lüften usw. Alles schön und gut, die saßen da in ihren Daunenmänteln da drin. Also ich weiß nicht, wie viel wir dann infiziert haben dann dadurch, ja, die dann doch Schnupfen, Husten, Heiserkeit gekriegt haben. Also das ist schon... nicht optimal… Man kann auf der einen Seite sagen, wir sind so, jetzt immer auch durchgekommen. Naja...] mehr schlecht als recht. (*D*, O2)

Der Kliniker wird den Patienten erst dann isolieren, wenn er ein Ergebnis hat. [Ja, weil die Maßgabe vom Haus ist, der Patient hat Durchfall, hat einen respiratorisches Infekt, der muss einzeln gelegt werden. Das geht in der Praxis häufig nicht.] (*L*1)

Ich denke, es ist ja bisher gut gegangen alles, wieso soll man da jetzt viel ändern. (*P*, D1)

Wenn ich nur Ambulanz und keine Intensivpatienten oder hämatologische Patienten habe, kann man die durchaus ohne führen. (*L*2)

[Sowas wär wahrscheinlich dann in Einzelfällen nett, aber tatsächlich sonst wenig-...] wahrscheinlich wirds wenig bringen. (*D*, O1)

[… natürlich ist das ein deutlicher Mehraufwand und] da ist natürlich die Resonanz von den (lacht) Kollegen immer eher, ja, nicht so gut sag ich mal so. (*H*, P)

Es ist halt nochmal ein zusätzlicher Personalaufwand mit Leuten die ich eh nicht hab. (*D*, O1)

[Also ich glaub allgemein, also jetzt nicht nur zu diesem PCR-Schnelltest, ich glaub allgemeint ist es schon eher so, dass es fürs Personal eine] deutliche Mehrbelastung [ist seit COVID ist. Und da ist halt natürlich jeder-... so könnte ichs mir vorstellen, ich weiß nicht, aber da ist natürlich dann jeder Mehraufwand, wenn ich erst halt einen Schnelltest machen muss, dann muss ich noch nen PCR-Schnelltest machen und dann vielleicht noch nen PCR-Test ins Labor schicken.] (*H*, P)

Wenn mittags komplett die Ambulanz voll ist und man eigentlich gar nicht weiß, wo vorne oder hinten ist. Und dann noch jemand kommt und so nen Test will, ist nicht der Test oder die Erfindung an sich das Problem, sondern halt einfach weil man weiß oder sich denkt, boah jetzt muss ich das auch noch machen. (*H*, E)

Also an sich, wenn man das als Standard nehmen würde und das halt dann gar nicht mehr ins Labor schicken würde, dann würde das auf jeden Fall Zeitersparnis. (*H*, P)

Man muss ja wirtschaftlich denken. Man hat eine Verantwortung dem Sozialstaat gegenüber. (*L*2)

Das Gerät ist sozusagen im Moment noch eine kostenneutrale Leihgabe. (*D*, D1)

Das ist die andere Frage, ob es eine Kostenübernahme gibt, was bezahlt wird von den Krankenkassen. Geräteanschaffung wahrscheinlich. Weniger Materialverbrauch, da wird es ja wahrscheinlich dann wie üblich irgendeine Ziffer geben. (*D*, D2)

Also da... ja, ist unsere Politik oder unser Gesundheitswesen nicht so einig, was sie wollen. Wollen sie Gesundheit oder wollen sie Geld? (*D*, D1)

Also Idealfall Testung in einem speziellen Raum, den wir dafür schon seit zwei Jahren jetzt eigentlich vorgesehen haben. Und real in einem großen Teil der Fälle, testen wir dann halt schon während der Dialyse. (*D*, D1)

[Klare Zuständigkeiten] sind wichtig aufgrund von Interdisziplinarität auch aufgrund von Absprachen, um Arbeitsprozesse auch flüssiger zu gestalten und auch Personalressourcen, ja, effektiv einzusetzen. (*D*, P)

]Es ist so, dass wir Gerätebeauftragte haben, die mit dem Gerät sich] gut auskennen auch die Materialien kennen, wie man es anwenden muss, Kontakte natürlich auch zum, zum Kundenservice dann auch haben wenn mal was nicht funktioniert. (*D*, P)

[-... es ist] idiotensicher [eigentlich. So am Anfang, gut, man muss es halt ein zwei Mal gemacht haben, aber an für sich ist es] selbsterklärend. (*H*, P)

Es fallen dann natürlich wieder Überstunden an. So ist das dann halt. (*H*, E)

Aus meiner Sicht ein No-Go. Es sollte medizinisch geschultes Personal sein. (*L*2)

Also ich fühl mich jetzt sicher [bei der Anwendung]. (*H*, P)

... ja-... könnte ich mir halt vorstellen, wenn es irgendwelche Fehlerquellen gibt, dass natürlich das dann immer weitergegeben wird. Wenn man so eingearbeitet wird. [...] Das denke ich wär schon auch nicht schlecht, wenn man da vielleicht auch öfters irgendwie Termine oder Schulungen dazu hätte, dass auch jeder quasi eine adäquate Schulung [...] gekriegt hat. (*H*, P)

Weil dann nicht wild durcheinander getestet wird, sondern man wirklich ne klare Leitlinie hat, wo man weiß, okay, das und das muss vorliegen, dass dieser Test eben gerechtfertigt ist. Damit es halt eben auch einen optimalen Nutzen erreichen kann. (*H*, E)

[Dass die auf die Idee kommen das Gerät steht jetzt da, ich kann das überall einfach anordnen und dass wir auch tatsächlich gemeinsam ein bisschen geguckt haben, uns da zu] schützen. (*D*, E)

Also die Vorgaben sind auch von der Taskforce natürlich [...] quasi gegeben und dem..., ja, muss man sich dann halt so fügen. (*H*, E)

Das wird ja oft, oder ist ja mit vielen Sachen so, dass halt Leute entscheiden, die nicht da arbeiten oder damit arbeiten. (*H*, E)

[Auf jeden Fall dauerhaft.] Ich finde es super, wenn das hier steht und wenn man es mit Sinn und Verstand einsetzt, kann man das weiter auf jeden Fall nutzen. (*D*, D1)

Weil ich mir denke Pandemien werden immer kommen, wenn man immer mehr dazu nimmt, irgendwann muss es auch mal gut sein. (*H*, E)

Ich weiß halt nicht, was die Zukunft bringt. [Ob es wieder mehr wird oder weniger.] (*P*, O2)

Ich gehe davon aus, dass die Leute wissen, was sie tun, und wenn die sagen, jetzt ist es nötig, dann ist es okay. (*P*, O2)

[Der ist in unserem Falle] überschaubar. [Dadurch-... wir machen grundsätzlich einen Schnelltest und einen klassischen PCR-Test. Der] Handgriff [findet ohnehin statt. Und die POCT-Variante ist relativ selten dann doch. Also das ist nicht der Mehraufwand, der zu irgendwelchem Unmut oder einer wirklichen Mehrbelastungen führt] (*D*, D1)

[ich bin auch […] Gutachter für das Qualitätsmanagement […]] von daher wusste ich also darüber zu jedem Zeitpunkt gut Bescheid um die Qualität der Untersuchungen an diesen Geräten und konnte auch gut Rückmeldung an die Task Force geben. (*L*1)

[Aber wir haben uns schon auch] aktiv eingebracht. [Ich glaube, wenn man es hätte laufen lassen. Also ich glaube, wir hatten eine ganz gute Position in der Covid-Sache, um uns da einzubringen. Die normale Krankenhaushierarchie hätte uns da sicher nicht so eingebunden.] (*D*, E)

[Es hat] eine gewisse Flexibilität auch Dinge nochmal anzupassen in der ersten Zeit. (*D*, E)

[Genau, wir haben diese Disketten, die testen können, eben auf Influenza A und B, auf RSV und auf Covid. Mit] einem Abstrich [und Test können wir alles mit abdecken. Das war sehr hilfreich jetzt über die Wintermonate.] (*H*, D1)
